# Structural Insights into the Azole Resistance of the *Candida albicans* Darlington Strain Using *Saccharomyces cerevisiae* Lanosterol 14α-Demethylase as a Surrogate

**DOI:** 10.3390/jof7110897

**Published:** 2021-10-24

**Authors:** Danyon O. Graham, Rajni K. Wilson, Yasmeen N. Ruma, Mikhail V. Keniya, Joel D. A. Tyndall, Brian C. Monk

**Affiliations:** 1 Sir John Walsh Research Institute, Faculty of Dentistry, University of Otago, PO Box 56, Dunedin 9054, New Zealand; danyon.graham@gmail.com (D.O.G.); rajni.wilson@otago.ac.nz (R.K.W.); yasmeen.ruma@otago.ac.nz (Y.N.R.); mikhail.keniya@otago.ac.nz (M.V.K.); 2 School of Pharmacy, University of Otago, PO Box 56, Dunedin 9054, New Zealand; joel.tyndall@otago.ac.nz (J.D.A.T.)

**Keywords:** azole resistance, *Candida albicans*, Darlington strain, *Saccharomyces cerevisiae*, CYP51, lanosterol 14α-methylase, crystal structures

## Abstract

Target-based azole resistance in *Candida albicans* involves overexpression of the *ERG11* gene encoding lanosterol 14α-demethylase (LDM), and/or the presence of single or multiple mutations in this enzyme. Overexpression of *Candida albicans* LDM (CaLDM) Y132H I471T by the Darlington strain strongly increased resistance to the short-tailed azoles fluconazole and voriconazole, and weakly increased resistance to the longer-tailed azoles VT-1161, itraconazole and posaconazole. We have used, as surrogates, structurally aligned mutations in recombinant hexahistidine-tagged full-length *Saccharomyces cerevisiae* LDM6×His (ScLDM6×His) to elucidate how differential susceptibility to azole drugs is conferred by LDM of the *C. albicans* Darlington strain. The mutations Y140H and I471T were introduced, either alone or in combination, into ScLDM6×His via overexpression of the recombinant enzyme from the *PDR5* locus of an azole hypersensitive strain of *S. cerevisiae*. Phenotypes and high-resolution X-ray crystal structures were determined for the surrogate enzymes in complex with representative short-tailed (voriconazole) and long-tailed (itraconazole) triazoles. The preferential high-level resistance to short-tailed azoles conferred by the ScLDM Y140H I471T mutant required both mutations, despite the I471T mutation conferring only a slight increase in resistance. Crystal structures did not detect changes in the position/tilt of the heme co-factor of wild-type ScLDM, I471T and Y140H single mutants, or the Y140H I471T double-mutant. The mutant threonine sidechain in the Darlington strain CaLDM perturbs the environment of the neighboring C-helix, affects the electronic environment of the heme, and may, via differences in closure of the neck of the substrate entry channel, increase preferential competition between lanosterol and short-tailed azole drugs.

## 1. Introduction

Superficial fungal infections affect approximately a billion people worldwide, and are usually readily treated. In contrast, mortality rates of 30–95% for invasive fungal infections result in almost 1.5 million deaths per annum [1]. *Candida albicans* is estimated to be responsible for approximately 40,000 of these deaths, with resistance to antifungal drugs becoming increasingly problematic. Fungal phytopathogens can compromise food security by reducing the yields of major crops such as wheat, rice, and soybeans, spoiling fruit such as bananas, apples, and grapes, and due to carcinogenic mycotoxins contaminating crops such as peanuts [2,3]. Human population growth and climate change require that food is produced efficiently, with the ravages caused by phytopathogens minimized. The identification of effective antifungals that are not subject to drug resistance is needed urgently by both medicine and agriculture.

*C. albicans* is usually a harmless commensal of the oropharynx and the gastrointestinal and reproductive tracts of humans [4,5]. Immunodeficiency and immunosuppression increase the risk of invasive infection by *C. albicans*, especially among premature babies and the elderly, cancer patients, recipients of organ transplants, and those with acquired immunodeficiency syndrome (AIDS) [6,7,8,9,10]. Intrinsic or acquired resistance to antifungal agents are problems that can compromise prophylaxis, therapies, and significantly increases the cost of hospitalization [10]. Furthermore, acquired resistance to almost every antifungal agent used to treat crops has emerged within a few seasons of their deployment, significantly increasing the cost of agriculture and the risk of pesticide side effects on the environment [3].

**Figure 1:**
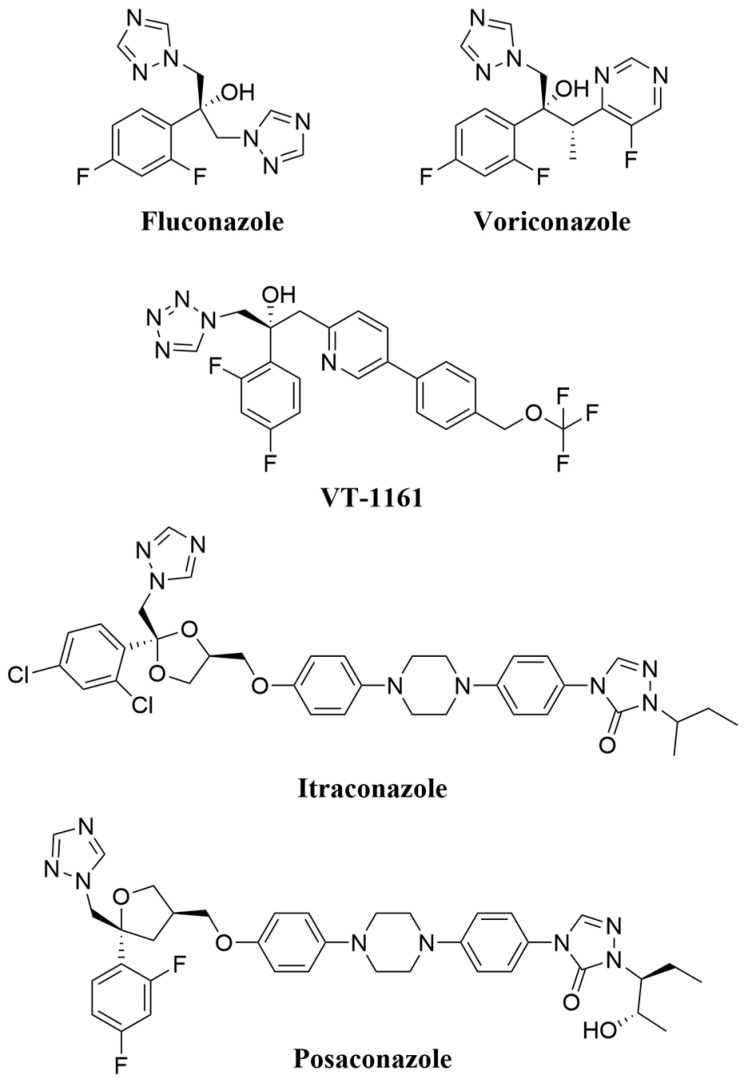
Chemical structures of short- (FLC and VCZ), medium- (VT-1161), and long-tailed (ITC and PCZ) azoles. FLC, VCZ and VT-1161 contain a tertiary alcohol that forms a water-mediated hydrogen bond network to tyrosine 140 in ScLDM6×His. ITC and PCZ do not contain a comparable hydroxyl group.

The azole antifungals (Figure 1) inhibit sterol 14α-demethylase (CYP51, SDM), thereby preventing biosynthesis of the essential and fungal specific sterol ergosterol, and resulting in the collateral synthesis of toxic sterols [11,12,13]. The triazole antifungals are inexpensive, orally available, and usually well-tolerated by patients. They are the most widely used class of antifungals for prophylaxis and treatment of invasive fungal infections, particularly where economic constraints, a more limited spectrum of action, and poor bioavailability restrict therapy with echinocandins [14,15,16,17]. Triazole antifungals are estimated to account for ~20% of the global market share of the systemic fungicides used in agriculture [3].

Although the azole agrochemicals are usually described as fungicidal, the triazole antifungals used in medicine are fungistatic. Importantly, triazole prophylaxis in immunocompromised patients, prolonged treatment regimens, and their widespread use in agriculture can select for triazole resistant fungal strains [10,18,19,20]. Point mutations in the *C. albicans ERG11* gene are a major mechanism of resistance, with over 140 different amino acid substitutions reported in fluconazole (FLC) resistant clinical isolates, although only a few of these mutations have been correlated definitively with azole resistance [21]. A common and clinically relevant mutation in *C. albicans* lanosterol 14α-demethylase (CaLDM) confers resistance to FLC and voriconazole (VCZ) by substituting the tyrosine at position 132 within the substrate binding site with phenylalanine (Y132F) or histidine (Y132H) [22,23,24,25,26,27,28,29,30,31]. Homologous mutations are found in the *ERG11* genes of other fungal pathogens of humans and plants [2,3,32]. Compared with single site mutations, multisite mutations in LDM can significantly increase FLC resistance [21]. The I471T mutation has been found only in the presence of the Y132H mutation in CaLDM, i.e., the Y132H I471T combination found in the highly FLC resistant Darlington strain of *C. albicans* (NCPF 3310) [26,29]. The Darlington strain of *C. albicans* was isolated from a child with chronic mucocutaneous candidiasis, who became unresponsive to long-term treatment with ketoconazole [33]. The strain appears to have an altered sterol content, reduced Δ^5,6^-sterol desaturase (Erg3) activity, and azole resistance unlikely to be entirely due to energy-dependent drug efflux [34,35,36]. Azole resistance can occur via various mechanisms in addition to target site mutations in CaLDM. These include the overexpression of *CaERG11* and drug efflux pumps [37,38] due to gain-of-function mutations in the transcription factors (Upc2, Mrr1 and Tac1), and aneuploidy and isochromosome formation in a region of chromosome 5 which encodes Erg11 and the Tac1 transcriptional regulator that affects expression of the CaCdr1 drug efflux pump [39]. However, these mechanisms have yet to be demonstrated for the Darlington strain. Replacement of one of the two *ERG11* alleles in an azole-susceptible isolate of *C. albicans* with a copy encoding the Y132H and I471T mutations confers increased FLC resistance [26]. It has been suggested that FLC resistance due to the I471T mutation results from a combination of increased catalytic turnover, increased affinity for substrate, and a reduced affinity for FLC in the presence of substrate [40], but how this happens is not understood. A similar combination of mutations (Y131F I475T) is found in the SDM of some azole resistant strains of the phytopathogen *Phakopspora pachyrhizi* [41]. This double mutation has been reported in approximately 15% of isolates from soybean plants in Brazil, and conferred substantial resistance (~100-fold) to the azole agrochemicals cyproconazole, epoxiconazole, metconazole, and tebuconazole, together with less than a 2-fold induction of *ERG11* mRNA expression [41].

X-ray crystal structures of recombinant full-length C-terminal hexahistidine-tagged wild-type *Saccharomyces cerevisiae* LDM (ScLDM6×His) show the phenolic hydroxyl of Y140 (*S. cerevisiae* numbering, equivalent to Y132 in CaLDM and Y131 in *P. pachyrhizi* SDM) hydrogen bonds with the heme ring C propionate, and forms a water-mediated hydrogen bond network with the tertiary alcohol of the short-tailed triazoles FLC and VCZ (PDB ID: 4WMZ and 5HS1, respectively) [42,43], and the medium-tailed tetrazole VT-1161 [44]. Mutation of Y140 to F140 or H140 disrupts these interactions between the enzyme and FLC (PDB ID: 4ZE3 and 4ZDZ, respectively), and reduces susceptibility to the drug [43]. In contrast, the effect of the I471T mutation is likely to be indirect. Modeling the I471T mutation inserted into the crystal structure of full-length CaLDM in complex with itraconazole (ITC) (PDB ID: 5V5Z) [45] using Pymol suggests that the I471T sidechain occurs into a hydrophobic pocket, adjacent to the heme ring C and beside helix C. In particular, the T sidechain oxygen is within 4.2 Å of the heme pyrrole C ring methyl group, and within 5 Å of helix C K143 (βC and εC), A146 (βC), K147 (γC), and L150 (δC). The K143 sidechain amino group forms an ionic bond with the heme ring C propionate. In *Trypanosoma cruzi* and human CYP51, the structurally equivalent ionic contact is lost, and the K sidechain becomes surface exposed on lanosterol binding [46,47]. We hypothesize that introduction of the mutant hydrophilic I471T hydroxyl group into this hydrophobic environment significantly reduces local hydrophobic interactions, thereby increasing the opportunity for interaction with the cognate NADPH cytochrome P450 reductase, and encouraging residence in a substrate binding mode.

We have expressed ScLDM6×His as a surrogate for the Darlington LDM Y132H I471T mutant of *C. albicans*, and the related LDM Y131F I475T mutant of *P. pachyrhizi*. Mutant ScLDM6×His containing the Y140H and I471T mutations, either alone or in combination, were used to determine azole susceptibilities and novel X-ray crystal structures. We tested the hypothesis that the ScLDM Y140H I471T mutant has an altered heme environment, preventing the stable binding of short-tailed azoles. Structure-activity relationships obtained for the clinically relevant azole antifungals VCZ and ITC support the hypothesis that the binding of short-tailed azoles by LDM is not only directly affected by Y140F/H mutations in the active site, but also by the structurally remote I471T mutation modifying the environment of the heme, and potentially increased competition with the enzyme substrate lanosterol. In contrast, interactions of medium- to long-tailed azoles with the walls of the LDM substrate entry channel (SEC) stabilize drug binding, and mitigate the effects of the Y140F/H and I471T mutations in the active site.

## 2. Materials and Methods

### 2.1. Yeast Strains

Yeast strains used in this study (Supplementary Table S1) were prepared using the AD2Δ strain of *S. cerevisiae* as a host, using an established protocol [43]. AD2Δ is hypersensitive to xenobiotics due to the deletion of seven major ABC transporters, including those responsible for the efflux of azole drugs [48]. It is also deleted of the *PDR3* transcriptional regulator, but contains the gain-of-function *Pdr1-3* transcriptional regulator mutation that leads to constitutive overexpression from the *PDR5* locus.

### 2.2. Construction of ScLDM6×His Encoding Mutants

Mutant *ERG11* constructs were made using PCR-based site-directed mutagenesis of the wild-type *ScERG11-6×His* open reading frame (ORF), using the high fidelity KOD^+^ DNA polymerase (Novagen, Madison, WI, USA), and the primers listed in Supplementary Table S2. Mutations were introduced by amplifying wild-type *ScERG11-6×His* template DNA with *PDR5* flanking primers and complementary internal mutagenic primers that overlap at the site of mutation [43]. To create the surrogate Darlington enzyme ScLDM Y140H I471T, mutagenic primers encoding the I471T mutation were used with template DNA that encoded the Y140H mutation. Recombinant PCR was used to generate transformation cassettes that contained the entire *ScERG11-6×His* ORF, plus the *PGK* transcriptional terminator and a downstream *URA3* selection marker, flanked by *PDR5* upstream and downstream sequences. 

Transformation cassettes encoding the ScLDM Y140H or I471T mutations, or both mutations, were separated in 0.8% agarose gels, isolated using a gel purification kit (Qiagen Pty Ltd., Limburg, The Netherlands), and transformed into the *PDR5* locus of strain AD2Δ through homologous recombination, using the Alkali Cation Yeast Transformation kit (Qbiogene, Irvine, CA, USA) to create strains AD2Δ ScLDM Y140H, AD2Δ ScLDM I471T, and AD2Δ ScLDM Y140H I471T. Transformants were selected by incubation on SD-Ura agar dropout plates (Formedium, Hunstanton, UK) at 30 °C for 48–72 h. For each transformation, 12 transformants were cultured onto YP-glycerol agar to eliminate petite colonies. Genomic DNA of selected transformants was extracted using the Y-DER reagent kit (Thermo Fisher, Waltham, MA, USA). The *PDR5* locus was PCR amplified using KOD^+^ DNA polymerase (Novagen, Madison, WI, USA), sequenced at the Genetic Analysis Service, University of Otago, New Zealand, and aligned to the wild-type *ScERG11* sequence using the BLAST algorithm to confirm the presence of the expected mutated LDM. 

The native *ScERG11* gene in yeast cells containing mutant *ScERG11-6×His* constructs in the *PDR5* locus was disrupted by homologous recombination with a cassette encoding the *His1* selection marker and sequences homologous with the regions bordering the endogenous *ScERG11* ORF to create strains AD3Δ ScErg11 Y140H, AD3Δ ScErg11 I471T and AD3Δ ScErg11Y140H I471T [43]. Transformants were selected on SD-His agar plates incubated at 30 °C for 48–72 h. Genomic DNA was extracted, and DNA sequencing as above was used to confirm deletion of the native *ERG11.* Strain AD3Δ ScErg11 (denoted as AD3Δ), which overexpresses wild-type ScLDM6×His from the *PDR5* locus and has the native *ScERG11* gene disrupted, was used as a control strain [43].

### 2.3. Drug Susceptibility Assays

Azole susceptibility was measured as the mean drug concentration required to inhibit 80% of yeast growth (MIC_80_) compared to a non-drug control, using a standard broth microdilution assay [37]. Serial dilutions of short- (FLC and VCZ), medium- (VT-1161), and long-tailed (ITC and PCZ) azole drugs, and the control drugs micafungin (MCF) and amphotericin B (AMB), were prepared in a 96-well microtiter plate in complete synthetic media lacking uracil, buffered to pH 6.8 [34]. Control and test cells were seeded at an optical density of 600 nm (OD_600_) of 0.005 (~1.5 × 10^4^ colony forming units). After incubation for 48 h at 30 °C with constant agitation (200 rpm), the OD_600 nm_ of each well was measured using a Synergy 2 multi-mode plate reader (BioTek Instruments, Winooski, VT, USA). The experiment was repeated three times (a total of nine measurements for the response of each strain to each drug). GraphPad Prism 5.0 software was used for statistical analysis, using a one-way analysis of variance with a Bonferroni post-hoc test. The statistical significance of comparisons was accepted when *p* < 0.05.

### 2.4. Agarose Disk Diffusion Assays

Agarose diffusion assays were carried out as described previously [49] using the Darlington strain of *C. albicans* (Pfizer Ltd., Sandwich, UK) and the control *C. albicans* database strain SC5314, which is not resistant to azoles. The Cdr1p-specific inhibitor RC21v3 and the CaMdr1p-specific inhibitor MCC1189 (MicroCombiChem, Wiesbaden, Germany), included in the assay medium at 6 µM, were used to investigate the contribution of efflux pumps to azole resistance in the Darlington strain [49,50,51]. 

#### Purification of ScErg11p6×His

Protein purification was carried out using established methods [42,43,52]. In brief, a 12 L culture for each control and test strain was grown overnight in 1 L volumes of YPD medium (1% [*w*/*v*] yeast extract [BD Difco], 2% [*w*/*v*] peptone [BD Difco], and 2% [*w*/*v*] D-glucose) in baffled 3 L flasks at 30 °C, with constant agitation (140 rpm). Cells harvested by centrifugation were broken by bead-beating and crude membrane fractions obtained by differential centrifugation, as previously described [52]. The protein concentration of the crude membranes was estimated using the Lowry assay, with bovine serum albumin (Thermo Fisher) used as the protein standard [53]. The crude membranes (1 g at 2.5 mg/mL) were detergent-solubilized using 10× critical micelle concentration (CMC) *n*-decyl-β-d-maltoside (DM) (Anatrace, Maumee, OH, USA) in buffer containing 10% (*w*/*v*) glycerol, 250 mM NaCl, 20 mM Tris pH 7.5, 0.5 mM phenylmethanesulfonyl fluoride (PMSF), and 1 EDTA-free protease inhibitor pill (Roche, Basel, Switzerland) per 300 mL. When the protein was to be used to assess in vitro binding affinity for the test azoles, detergent solubilized ScLDM6×His recovered after centrifugation was affinity purified using a nickel-nitrilotriacetic acid (Ni-NTA)-agarose matrix (Qiagen). The bound ScLDM6×His was eluted from the matrix using an affinity purification buffer containing 50 mM L-histidine [40]. The L-histidine was removed by washing the enzyme through centrifugal filtration, using a 50 kDa molecular-weight cut-off Amicon Ultra-4 filter (Sigma, Auckland, New Zealand).

For protein crystallization experiments, DM-solubilized crude membrane protein recovered after centrifugation was filtered, and loaded onto a 1 mL nickel Sepharose affinity purification column (HisTrap HP, GE Healthcare Life Sciences, Auckland, New Zealand) at 1 mL per min using an ÄKTA pure chromatography system (GE Healthcare Life Sciences) at 6 °C, controlled using UNICORN 7.0.2 software. The column was washed with 15 volumes of affinity purification buffer (10% (*w*/*v*) glycerol, 250 mM NaCl, 20 mM Tris pH 7.5, 0.5 mM PMSF, 6.4 mM DM, 20 mM imidazole, and 1 EDTA-free protease inhibitor pill per 300 mL). The bound ScLDM6×His was eluted from the column in reverse flow, using affinity purification buffer containing 200 mM imidazole. Once A_420nm_ = 100 mAU was reached, the next 2.5 mL eluted was redirected to a holding loop. The stored sample was purified by size exclusion chromatography (SEC), using a Superdex 200 10/300 GL column (GE Healthcare Life Sciences) equilibrated with purification buffer (10% *w*/*v* glycerol, 250 mM NaCl, 20 mM Tris pH 7.5, 0.5 mM PMSF, 6.4 mM DM, 20 mM imidazole, and 1 EDTA-free protease inhibitor pill per 300 mL at 6 °C, at a flow rate of 0.45 mL per min. The sample was loaded onto the SEC column in 3 mL, and eluted isocratically with 1.5 column volumes of purification buffer. The peak of the purified 62-kDa ScLDM×His was pooled and concentrated using a 50 kDa molecular-weight cut-off Amicon Ultra-4 centrifugal filter (Sigma), in the presence of 40 μM of either VCZ or ITC at 4 °C.

### 2.5. Quantitation of ScLDM6×His Expression

Crude membrane samples (15 µg of protein) were separated by SDS-PAGE on 8% polyacrylamide. Pre-stained protein standards (5 μL) (PageRuler Plus, Thermo Fisher, Auckland, New Zealand) were used to estimate the relative size of the proteins. Coomassie blue R250 stained gel bands were visualized using an Odyssey Fc imaging system (LI-COR, Lincoln, NE, USA), and the relative content of the 62 kDa band calculated using Image Studio Lite 5.2 (LI-COR, Lincoln, Nebraska, NE, USA), and normalized to the 99 kDa *S. cerevisiae* plasma membrane proton pump (ScPma1p) as a loading control.

Recombinant ScLDM6×His was detected and quantitated by electrotransfer of 15 µg crude membrane samples, separated by SDS-PAGE, to nitrocellulose membranes. The transfers were treated for 1 h at 25 °C in blocking buffer (1.5% *w*/*v* skim milk and 0.02% *v*/*v* Tween-20 in phosphate buffered saline (PBS)), and decorated with a 1:3000 dilution of a Roche mouse anti-6×His-peroxidase monoclonal antibody (Sigma) in blocking buffer for 1 h at 25 °C. The transfers were washed three times for 5 min in PBS containing 0.1% *v*/*v* Tween-20 at 25 °C, and incubated in detection solution (5.5 mg of Luminol in 60 µL of dimethyl sulfoxide (DMSO), 0.28 mg of p-coumaric acid in 10 µL of DMSO, 7.7 µL of 30% hydrogen peroxide, and 25 mL of 0.1 M Tris-HCL at pH 8.6). The chemiluminescence of the antibody-decorated protein bands was visualized using an Odyssey Fc imaging system (LI-COR). Grey values representing the relative amounts of the protein-6×His were calculated using Image Studio Lite (version 5.2) (LI-COR). 

### 2.6. Spectral Characterization of Purified ScLDM6×His

Spectral characterization of Ni-NTA purified ScLDM6×His was conducted using a Varian Cary 300 UV-visible spectrophotometer. The amounts of wild-type and mutant ScLDM6×His detected at ~420 nm were normalized by diluting samples in the SEC purification buffer to the same absorbance at 280 nm. 

### 2.7. In Vitro Binding Affinity of Test Azoles to ScLDM6×His

The concentration of functional ScLDM6×His purified by the nickel affinity method was quantitated using the standard carbon monoxide (CO) binding method [54]. Type II drug binding assays that measure absorbance changes due to ligand interactions with ScLDM6×His were performed for VCZ and ITC, using an established protocol [42,43]. The peak_429nm_-trough_410nm_ difference spectra plotted against azole concentration were used to generate drug binding curves. All calculations were performed using GraphPad Prism 5 Software.

### 2.8. Crystallization of ScLDM6×His, X-Ray Data Collection and Molecular Model Building

Crystal trials using nickel affinity and size exclusion chromatography purified ScLDM6×His containing either the Y140H, I471T, or Y140H I471T mutations in complex with either VCZ or ITC were set up using the hanging drop vapor diffusion method [52]. The reservoir solution contained 43–46% polyethylene glycol 400 (PEG 400), and 0.5 M glycine at a pH range of 9.1–9.6. The drops were set up as 1 µL volumes in a 1:1 ratio of reservoir solution and ~20 mg/mL of the purified protein in size exclusion chromatography buffer, using a Mosquito crystallization robot (TTP labtech). The plates were incubated at 16 °C in a Rock Imager (Formulatrix) at the Department of Biochemistry Crystallography Suite, University of Otago, and monitored weekly for crystal formation. Purified wild-type ScLDM6×His (from strain AD3Δ) in complex with either VCZ or ITC were used as a positive control [43].

Complete X-ray diffraction data sets were collected at 0.954 Å at the MX1 beamline of the Australian Synchrotron, using an ADSC Quantum 210r detector under a cryostream.

The diffraction data was processed in iMosflm or XDS from the CCP4 suite [55,56]. Molecular replacement was carried out using ScLDM6×His complexed with lanosterol (PDB ID: 4LXJ) as the template by using Phaser-MR from the Phenix software suite after removing the ligand from the structure [52,57,58]. Phenix.refine was used to refine the structure [59]. Between rounds of refinement, COOT was used to visualize the structure, and to manually modify the structure to better fit the observed electron density map [60]. Crystallographic information files for VCZ and ITC were obtained from the CCP4 online database, and the azoles were modeled into the appropriate electron density within the active site. Water molecules were modeled into the structure if the observed electron density was within hydrogen bonding distance (2.5–3.3 Å) to an appropriate partner. Data collection and refinement statistics are shown in Supplementary Table S3.

## 3. Results

### 3.1. The ScLDM Y140H Mutation Confers Azole Resistance to FLC, VCZ, VT-1161 but Not ITC and Posaconazole (PCZ) 

**Figure 2:**
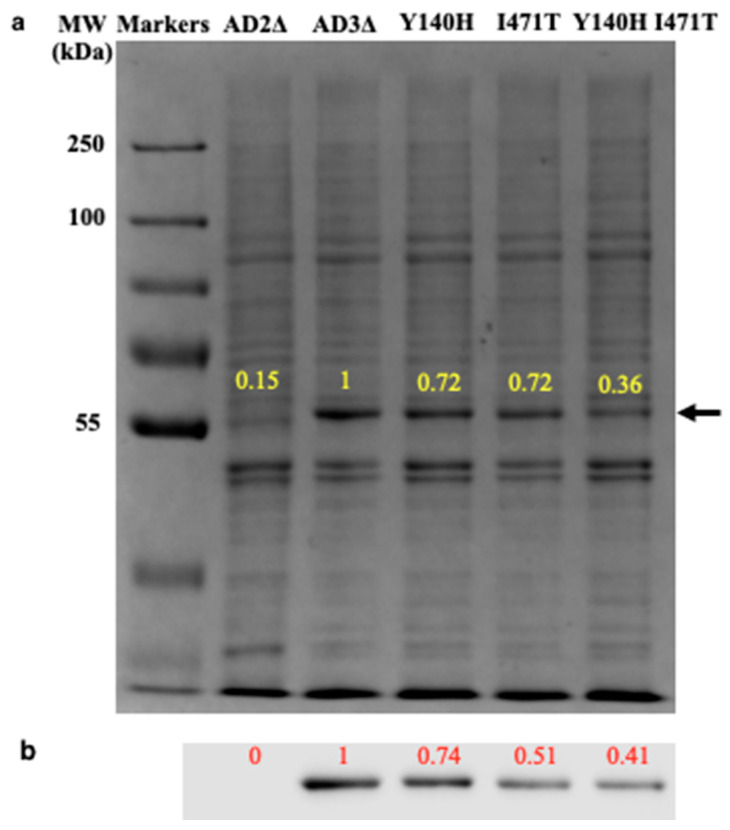
SDS-PAGE and western blot analysis of crude membrane fractions from control and test strains. (**a**) Coomassie blue R250-stained 8% acrylamide SDS-PAGE gel of samples of crude membranes (15 μg of protein) from strains overexpressing wild-type (AD3Δ) and mutant ScLDM6×His, with strain AD2Δ used as a negative control. The positive control AD3Δ ScLDM6×His band was assigned an arbitrary value of 1.00, and the intensity of the bands detected in test strains determined relative to this (yellow numbers). The black side arrow indicates the 62 kDa band containing ScLDM6×His; (**b**) Separated proteins electrotransferred onto nitrocellulose membranes were decorated with a mouse anti-6×His-peroxidase conjugated monoclonal antibody, and visualized using chemiluminescence. The band intensity for ScLDM6×His expression in strain AD3Δ was assigned an arbitrary value of 1.00, and the intensity of the bands detected in test strains determined was relative to this (red numbers). An Odyssey Fc (LI-COR) imaging system was used to visualize the Coomassie blue stained gel and the Western blot.

Strain AD3Δ ScErg11 (AD3Δ), which overexpresses ScLDM6×His from the *PDR5* locus and is deleted of the native LDM, conferred a 2- to 3-fold reduction in susceptibility to all azoles tested, compared with the control host strain AD2Δ, which overexpresses its native wild-type ScLDM at several-fold lower levels (Table 1 and Figure 2a). Furthermore, the overexpression of ScLDM6×His in strain AD3Δ did not modify susceptibility to the control antifungals micafungin (MCF) and amphotericin B (AMB), which do not target LDM.

Constitutive expression of hexahistidine-tagged recombinant LDM mutant enzymes (ScLDM6×His Y140H, ScLDM6×His I471T and ScLDM6×His Y140H I471T) from the *PDR5* locus supported normal growth in yeast cells with the native *ERG11* gene deleted. We therefore tested whether the Y140H and I471T mutations modify the expression of LDM and the in vitro binding of azole drugs. Coomassie blue staining of crude membrane preparations indicated that the recombinant mutant enzymes were overexpressed (Figure 2a). Their identities were confirmed by mass spectrometry of tryptic fragments of bands migrating at the molecular weight of 62 kDa predicted for the recombinant enzymes (Supplementary Figure S1). Western blots of the crude membrane fractions probed with an anti-His-tag antibody confirmed these observations and showed that ScLDM6×His Y140H, ScLDM6×His I471T, and ScLDM6×His Y140H I471T were expressed at 0.74, 0.51, and 0.41 of the wild-type ScLDM6×His, respectively (Figure 2b).

Strain AD3Δ ScErg11 Y140H, expressing ScLDM6×His Y140H, gave ~2-fold higher MIC_80_ values to the short-tailed azoles FLC and VCZ than strain AD3Δ ScErg11 (*p* < 0.001) (Table 1), despite expressing the mutant recombinant enzyme at 0.74 of the level of the overexpressed wild-type recombinant enzyme. 

VT-1161 is a tetrazole designed to induce reduced binding to human cytochrome P450 enzymes, and hence minimize drug interactions in the host [61,62]. This drug candidate is a potent inhibitor of *C. albicans* growth, and has completed phase IIb clinical trials for the treatment of onychomycosis of the toenail and recurrent and acute vulvovaginal candidiasis (ClinicalTrials.gov ID: NCT02267356, NCT02267382, NCT01891331, respectively), and is undergoing phase III clinical trials in several countries [44]. The mutant cells gave an MIC_80_ for VT-1161 1.5-fold higher than higher than AD3Δ cells (*p* < 0.001) (Table 1). When the relative amount of recombinant ScLDM6×His expressed was taken into account, ScLDM6×His Y140H appeared to confer 2.8-, 2.4-, and 2.1-fold resistance to FLC, VCZ, and VT-1161, respectively (Table 2).

Comparison of the published X-ray crystal structures of ScLDM6×His and ScLDM6×His Y140H in complex with FLC revealed that the Y140H mutation prevents formation of a water-mediated hydrogen bond between the phenolic hydroxyl of Y140 and the tertiary hydroxyl of FLC (PDB IDs: 4WMZ and 4ZE3, respectively) [43]. Despite this mutation reducing the affinity of ScLDM6×His for FLC and conferring drug resistance, FLC still maintains a water-mediated hydrogen bond network with the hydroxyl of Y126, and the main chain nitrogen of S382 via the non-coordinating triazole.

The X-ray crystal structure of ScLDM6×His in complex with VCZ (PDB ID: 5HS1) shows that the VCZ tertiary alcohol forms a water-mediated hydrogen bond network with Y140 and the heme ring D propionate. In addition, one nitrogen of the 5-fluoropyrimidine ring forms a water-mediated hydrogen bond network with the main chain nitrogen of H381, and the main chain carbonyls of S382 and M509. The X-ray crystal structure of ScLDM6×His Y140F in complex with VCZ (PDB ID: 4ZE0) shows F140 occupies less space in the active site than Y140 in the wild-type enzyme (PDB ID: 5HS1) [43]. An active-site water molecule, located 1.9 Å from the tertiary hydroxyl of VCZ, forms hydrogen bonds with VCZ and the heme ring D propionate, but not with Y140F. The water-mediated hydrogen bond network between VCZ and Y140, and a hydrogen bond between Y140 and the heme ring C propionate is lost due to the Y140F mutation. In addition, there is no water-mediated hydrogen bond network between 5-fluoropyrimidine ring and the main chain nitrogen of H381, and the main chain carbonyls of S382 and M509.

VT-1161 contains a tertiary alcohol comparable to that of FLC and VCZ within the linker between the inhibitor’s head and tail regions (Figure 1). The crystal structures of ScLDM6×His in complex with VT-1161, FLC, or VCZ (PDB IDs: 5UL0, 4WMZ, 5HS1) and the crystal structure of the catalytic domain of N-terminal truncated CaLDM in complex with VT-1161 (PDB ID: 5TZ1) show that the tertiary alcohol of VT-1161 interacts via a water molecule with the phenolic hydroxyl of ScLDM6×His Y140, and should interact with the structurally aligned CaLDM Y132 [42,43]. Thus, the ScLDM6×His Y140H mutation was expected to abolish interaction between the tertiary alcohol of VT-1161, FLC, or VCZ with ScLDM, conferring reduced susceptibility of whole cells to growth inhibition by these drugs. 

Cells overexpressing ScLDM6×His Y140H showed slightly increased susceptibility to the long-tailed azoles ITC and PCZ than AD3Δ cells (Table 1). Much of this increased susceptibility can be accounted for by the lower level of the ScLDM6×His Y140H enzyme (0.71 of the control enzyme) detected in crude membrane preparations, compared with preparations from AD3Δ cells (Table 2). ITC and PCZ lack the hydroxyl group of the short- and medium-tailed azoles, and instead contain a methoxy oxolane (PCZ) or methoxy dioxolane (ITC) linker that fills the space occupied by the active site water beside Y140 [43].

### 3.2. The ScLDM Y140H and I471T Mutations Synergistically Reduce Drug Susceptibility to FLC and VCZ but Not VT-1161, ITC or PCZ

Expression of ScLDM6×His I471T (strain AD3Δ ScErg11 I471T) increased susceptibility of *S. cerevisiae* cells to FLC (1.9-fold), VCZ (1.2-fold), VT-1161 (2.2-fold), ITC (1.4-fold), and PCZ (1.6-fold), but not to the control drugs MCF and AMB (Table 1). However, when the relative level of expression of the ScLDM6×His I471T was taken into account (0.51 of the control wild-type recombinant enzyme), the mutation had no effect or weakly reduced susceptibility (0.9- to 1.6-fold, respectively) to the azole drugs tested, compared with the control strain AD3Δ.

Strain AD3Δ ScErg11 Y140H I471T gave MIC_80_ values for both FLC and VCZ that were approximately 3-fold higher than for AD3Δ ScErg11 cells (*p* < 0.001) (Table 1), and 1.5-fold higher than for cells overexpressing the ScLDM6×His Y140H mutation (*p* < 0.001). Taking into account the relative expression of ScLDM6×His Y140H I471T (0.41 compared with wild-type ScLDM6×His), the mutant enzyme conferred strong resistance to VCZ and FLC (6.5- to 7.7-fold) (Table 2). In comparison with strains overexpressing wild-type ScLDM6×His, ScLDM6×His Y140H, or ScLDM6×His I471T mutations separately, the phenotype of the ScLDM6×His Y140H I471T indicates that the Y140H and I471T mutations acted synergistically in conferring azole resistance to these short-tailed azoles. In contrast, overexpression of ScLDM6×His Y140H I471T conferred weaker resistance to VT-1161, ITC, and PCZ, i.e., 2.2-, 1.8-, and 1.5-fold, respectively. The modest resistance to these drugs appeared to involve additive contributions by the Y140H and I471T mutations. There were no statistically significant differences between the MIC_80_s of the control drugs MCF and AMB for the various cell types tested (*p* > 0.05). 

Similar results were found previously for *S. cerevisiae* cells expressing the CaLDM Y132H and I471T mutations (*C. albicans* numbering), either alone or in combination [26]. Although it was found differently that the CaLDM I471T single mutation conferred slight resistance to FLC, the present study, together with the absence of reports of *C. albicans* clinical isolates with the I471T mutation without the concurrent presence of the Y132H mutation, suggests that a CaLDM I471T mutation is unlikely to confer selective advantage. 

### 3.3. Role of Efflux Pumps in Azole Resistance in the Darlington Strain

**Figure 3:**
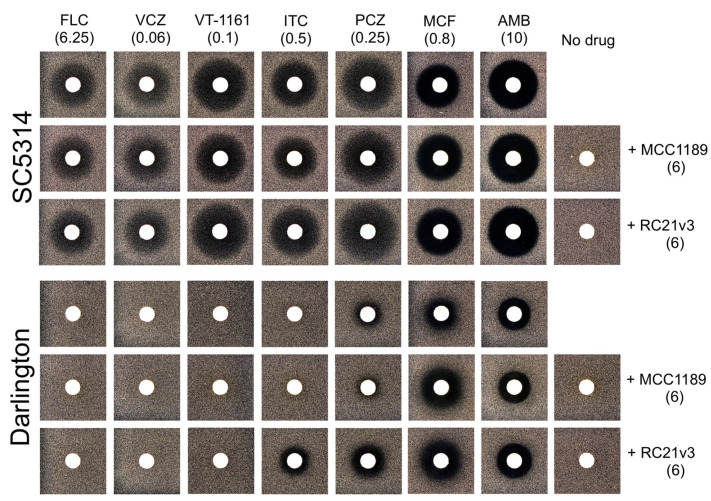
Agarose diffusion analysis of the interaction of control and azole drugs with wild-type *C. albicans* (SC5314), and the Darlington strain. The values in parentheses represent drug loadings in nanomoles.

Agarose diffusion drug susceptibility assays showed that the control *C. albicans* strain SC5314 was susceptible to FLC, VCZ, and VT-1161, while the *C. albicans* Darlington strain was resistant (Figure 3). Selective inhibition of the Major Facilitator Superfamily (MFS) drug efflux pump Mdr1 with MCC1189 [49] or the ABCG transporter Cdr1p with RC21v3 [50] did not alter the susceptibility of either the control or Darlington strain to FLC, VCZ, or VT-1161. Thus, the Cdr1 and Mdr1 efflux pumps are not implicated in the resistance of the Darlington strain to these azoles. The agarose diffusion assays confirmed the earlier finding that Darlington cells are resistant to ITC and PCZ [36]. Co-treatment of either SC5314 or Darlington cells with the Cdr1 inhibitor RC21v3 conferred increased susceptibility to both ITC and PCZ. Treatment with the Mdr1 inhibitor MCC1189 had no effect on susceptibility. These results indicate that efflux of long-tailed azoles through Cdr1, but not Mdr1, contributes to resistance in the Darlington strain. However, this conclusion maintains the caveats that MCC1189 is a substrate of Cdr1, and that PCZ is a poor substrate of CaMdr1 [49].

### 3.4. Electronic Properties of the Heme of Affinity Purified ScLDM6×His I471T and ScLDM6×His Y140H I471T

**Figure 4:**
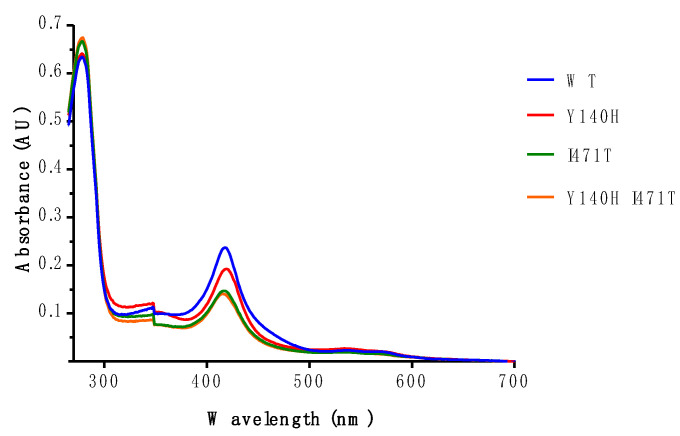
The absolute absorbance spectrum of apo-ScLDM6×His is modified by the Y140H, I471T, and Y140H I471T mutations.

**Figure 5:**
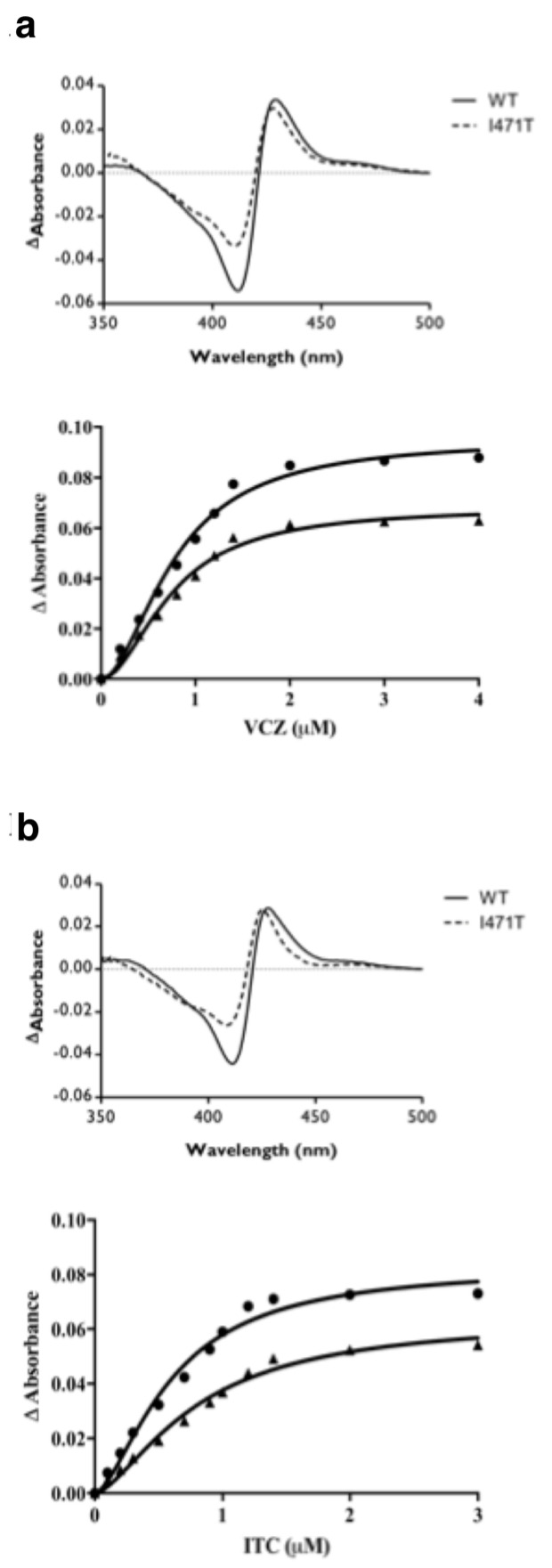
The I471T mutation affects binding of VCZ and ITC to affinity-purified ScLDM6×His. Difference spectra obtained in the presence of saturating (**a**) VCZ (3 µM) and (**b**) ITC (3 µM) with 2 µM ScLDM6×His wild-type and ScLDM6×His I471T are shown on the top of each figure, and the curves for type II binding saturation are shown on the bottom with ScLDM6×His wild-type (circles) and ScLDM6×His I471T (triangles).

Samples of recombinant wild-type and mutant ScLDM6×His were purified by Ni-NTA affinity chromatography, and characterized through spectrophotometry (Figure 4 and Figure 5). The absorbance spectra of the preparations, when normalized by their absorbance at 280 nm, differed significantly, but all showed heme Soret peaks at 418, 419, 417, and 416 nm for ScLDM6×His wild-type, Y140H, I471T, and Y140H I471T mutant enzymes, respectively (Figure 4). For comparable amounts of ScLDM6×His protein (detected at 280 nm), the maximum height of the Soret peak of the mutants was ~80% of the wild-type value for the Y140H mutant, and ~56% of the wild-type value for the Y471T and the Y140 I471T mutants. Thus, both mutations appear to affect the electronic environment of the heme, and this effect is strongest for the I471T mutation. The presence of the I471T mutation reduced the spectral response due to type II binding of VCZ and ITC, with the major difference observed in the trough region of the difference spectrum (Figure 5 and Supplementary Figure S2). Despite these changes, the type II binding curves showed that both VCZ and ITC bound tightly to the wild-type and I471T mutant enzymes, with each drug giving IC_50_ values of ~0.6–0.8 µM in the presence of 2 µM enzyme (Supplementary Table S4).

### 3.5. X-ray Crystal Structures of ScLDM6×His Y140H, ScLDM6×His I471T and ScLDM6×His Y140H I471T in Complex with Azole Drugs

**Figure 6:**
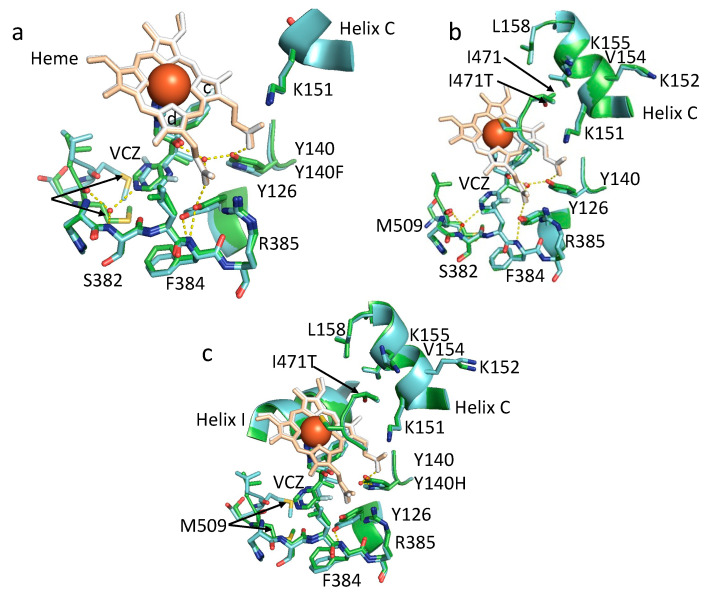
Comparison of the active sites of ScLDM6×His in complex with VCZ. (**a**) ScLDM6×His (carbons in green, heme in wheat, iron as large red sphere) compared with ScLDM6×His Y140H (carbons in azure, heme in white). The hydrogen bond network (yellow dashes) of ScLDM6×His is shown. The LDM Y140H mutation results in the absence of water molecules (small red spheres) within the active site, and the loss of the 2 water-mediated hydrogen bond networks involving VCZ. In ScLDM6×His Y140H, the absence of a water-mediated hydrogen bond involving VCZ and the main chain carbonyl of the M509 gives a significant displacement of this amino acid; (**b**) ScLDM6×His (carbons in green, heme in wheat) compared with ScLDM6×His I471T (carbons in azure, heme in white). The hydrogen bond networks of ScLDM6×His I471T but not ScLDM6×His are shown. The water-mediated hydrogen bond networks found in ScLDM6×His (Figure 6a) are maintained in ScLDM6×His I471T. The hydroxyl of I471T projects into the hydrophobic environment involving the carbon sidechains of K151, V154, K155, and L156 in helix C; (**c**) ScLDM6×His I471T (carbons in green, heme in wheat) compared with ScLDM6×His Y140H I471T (carbons in azure, heme in white). The hydrogen bond network of ScLDM6×His I471T is not shown. In ScLDM6×His Y140H I471T, the single water in the active site is located 3.6 Å from the tertiary alcohol of VCZ, and forms a hydrogen bond network with one nitrogen of the histidine imidazole and the heme ring.

Boat-shaped crystals of Ni-NTA affinity and size exclusion chromatography (SEC) purified ScLDM6×His Y140H in complex with VCZ formed after 15 days in 46% PEG, 0.5 M glycine pH 9.6. Boat-shaped crystals were also obtained using Ni-NTA affinity and SEC purified ScErg11p6×His I471T mutant in complex with either ITC or VCZ. These formed within one week across all experimental conditions tested (43–46% PEG 400 and 0.5 M glycine, pH 9.1–9.6). Similar crystals of ScErg11p6×His Y140H I471T in complex with VCZ formed after 20 days of incubation under a single experimental condition (46% PEG 400 pH 9.2). Crystals of ScLDM6×His Y140H in complex with VCZ diffracted to a resolution of 1.98 Å and ScLDM6×His I471T in complex with VCZ or ITC diffracted to resolutions of 2.4 and 2.1 Å, respectively, while ScLDM6×His Y140H I471T in complex with VCZ diffracted to a resolution of 2.9 Å (Supplementary Table S3, PDB IDs: 7RY8, 7RY9, 7RYA, 7RYB). The electron densities of the Y140H and I471T residues allow their conformations to be modelled in each relevant structure (Figure 6). 

Structural alignment of the X-ray crystal structure of ScLDM6×His in complex with VCZ (PDB ID: 5HS1) and ScLDM6×His Y140H in complex with VCZ (PDB ID: 7RY8) shows that H140, like F140, occupies less space in the active site than Y140. However, no active site water molecule was detected within 5 Å of VCZ in this 1.9 Å resolution structure, and the VCZ was displaced slightly towards H140 and helix I by <0.5 Å. Thus, there are no water-mediated hydrogen bond networks involving the tertiary alcohol of VCZ and Y140H and the heme ring D propionate, or between the 5-fluoropyrimidine ring and the main chain nitrogen of S382 and the main chain carbonyl of M509, as was found with the wild-type enzyme. The significantly different disposition of M509 between the wild-type and Y140H structures may be due to the absence of this water-mediated hydrogen bond network. There is also a significant impact on the disposition of Y126, which neighbors Y140/H, as the 5-fluoropyrimidine ring of VCZ was flipped 180 degrees between the wild-type and Y140H mutant structure. While the Y126 sidechain hydroxyl retained a hydrogen bond with the main chain amide of F384 in ScLDM6×His Y140H, a hydrogen bond with the heme ring D propionate was not formed. 

Structural alignment of the X-ray crystal structure of ScLDM6×His in complex with VCZ (PDB ID: 5HS1) with ScLDM6×His I471T in complex with VCZ (PDB ID: 7RY9) showed that the hydroxyl group introduced with the I471T mutation reached across the edge of the heme, and reduced the hydrophobic contacts in the corner of the active site beside helix I and the carbon-rich sidechain of K151 in helix C (Figure 6b). Otherwise, the two structures appeared identical and without loss of hydrogen bond interactions in the vicinity of the VCZ. However, modification of the environment near K151 is consistent with changes in the electronic environment of the heme detected in the spectral analysis and the azole binding studies shown in Figure 4 and Figure 5, respectively.

Structural alignment of the X-ray crystal structures of ScLDM6×His I471T in complex with VCZ and ScLDM6×His Y140H I471T in complex with VCZ (PDB ID: 7RYB) showed that the I471T sidechain in the double-mutant enzyme behaved identically to its counterpart in the I471T enzyme (Figure 6c). The VCZ in the double mutant showed the same configuration as in ScLDM6×His or ScLDM6×His I471T in complex with VCZ, but was slightly displaced towards H140 and more towards helix I (by ~0.7 Å) than in ScLDM6×His Y140H. The structure of ScLDM6×His Y140H I471T in complex with the VCZ structure also included a single water molecule, 3.6 Å from the tertiary alcohol of VCZ, that formed hydrogen bonds with the heme ring C propionate and a nitrogen of the histidine imidazole ring, but not VCZ. The structure showing the absence of a hydrogen bond between the tertiary alcohol of VCZ and the water is illustrated in Supplementary Figure S3. As was also observed in the structures of ScLDM6×His Y140F/H in complex with VCZ (PDB IDs: 4ZE0 and 7RY8) [43], the conformation of Y126 was altered in the Y140H I471T double-mutant structure such that its phenolic hydroxyl retains a hydrogen bond to the main chain N atom of F384, but no longer forms a hydrogen bond with the heme ring D propionate found in the wild-type and I471T single-mutant structures (Figure 6c). Distances between the sidechains of the structurally aligned residues K143, A156, K147, and L150 in I471 in wild-type CaLDM (PDB ID: 5V5Z) in complex with ITC and K151, V154, K155, L158, and I471T in ScLDM Y140H I471T in complex with VCZ (PDB ID: 7RYB) are all within 4–5 Å. Of these residues, K151 and V154 are slightly closer (4.5 Å), while K155 and L158 are slightly further (5 Å) from the I471T hydroxyl in ScLDM. In CaLDM, K143 and A146 are slightly further (4.9–5 Å), and K147 and L150 are slightly closer (4–4.6 Å) to I471. In the ScLDM surrogates, similar to K143 in CaLDM, K151 makes ionic contact with the heme ring C propionate (Figure 6a) and hydrogen bonds with main chain carbonyls of R467 and R469, which are adjacent to the C470 liganded with the heme iron. Similarly, R385 in the ScLDM surrogate retains its position and ionic contact with the heme ring D propionate. While the ionic interactions of the heme with K151 and R385 were retained in ScLDM6×His Y140H, the loss of hydrogen bonds between Y140 and the heme ring C propionate, and between Y126 and the heme ring D propionate may be expected to affect the electronic status of the heme. This interpretation is consistent with the slight (1 nm) red shift from 417 nm to 418 nm in the main Soret peak of ScLDM6×His Y140H (Figure 4). Furthermore, consistent with functional modification of the heme, the insertion of I471T into the hydrophobic environment near K151 gave blue shifts to 416 nm in both ScLDM6×His I471T and ScLDM6×His Y140H I471T preparations, as well as lower spectral responses to the binding of VCZ by ScLDM6×His I471T (Figure 4 and Figure 5). Despite these changes, the position of the heme cofactor and the amino acid backbone of residue 471 were not detectably different between the wild-type, Y140H or I471T single-, and Y140H + I471T double-mutant crystal structures of ScLDM6×His in complex with VCZ (Figure 6), or for the I471T mutant enzymes in complex with ITC (Figure S4). 

Studies with *Trypanosoma cruzi* human CYP51 show that the binding of the substrate lanosterol causes a small rotation of helix C [46,47]. This ablates a key ionic interaction with the heme ring C propionate, although in the ScLDM surrogate K151 would still interact with the main chain carbonyl of R467 in the fungal specific loop (FSL) via water-mediated hydrogen bonds. This subtle conformational change in helix C would enable K151, together with several basic groups, to interact more favorably with their cognate NADPH-cytochrome P450 reductase. The binding of the substrate also modifies the conformation of helix I, with an altered interaction at its N-terminal end, with helix G contributing to the opening and closure of the SEC mediated by the F-G helix. In contrast, it was not apparent how the Y140H and I471T mutations interact to confer preferential high-level resistance to the short-tailed azole VCZ, which appears to bind in the active site in comparable conformations in the Y140H and Y140H I471T enzymes. As the binding of the short-tailed azole drugs in ScLDM Y140H is more strongly dependent on interaction with the heme iron and hydrophobic contacts within the active site than for the long-tailed azole drugs which occupy additional sites in the SEC, we hypothesized that the I471T mutation confers greater opportunity for the enzyme’s substrate lanosterol to compete with azole drugs within the active site.

The lower resolution (2.9 Å) of the ScLDM6×His Y140H + I471T structure may have limited the observation of discrete structural changes, such as the location of key water molecules. However, alignment of the X-ray crystal structures of wild-type ScLDM6×His (PDB ID: 5EQB) and ScLDM6×His I471T (PDB ID: 7RYA) in complex with ITC not only showed that the ITC ligand adopted essentially identical conformations in both complexes, but also indicated the presence of a water molecule 2.6 Å from the hydroxyl of I471T that is not found in the wild-type structure (Supplementary Figure S4). This is of special interest, since the water forms a hydrogen bond network with the main chain carbonyl of K151. A more extensive electron density observed between T471 and the FSL may indicate the presence of molecules such as crystallization buffer components, e.g., PEG, the detergent DM used for protein solubilization, or a biological molecule carried through from the expression system. The water molecule and the additional electron density found near I471T were not observed in the structures of complexes of VCZ with ScLDM6×His Y140H, ScLDM6×His I471T, or ScLDM6×His Y140H I471T. The absence of a water molecule in the 1.9 Å resolution ScLDM6×His Y140H structure in the hydrophobic environment adjacent to I471 is not surprising, and this may not be detectable in the ScLDM6× Y140H I471T structure due to its lower resolution (2.9 Å). The capture of the water molecule by ScLDM6×His I471T complexed with ITC but not the complex with VCZ may have implications for catalytic efficiency. 

**Figure 7:**
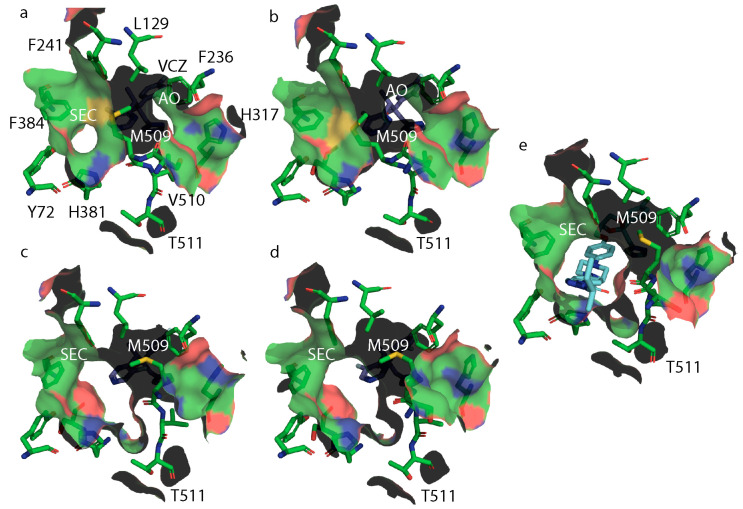
Surface structures at the neck of the substrate entry channel in ScLDM6×His and its mutants in complex with VCZ or ITC. (**a**) ScLDM6×His (WT) + VCZ (PDB ID: 5HS1); (**b**) ScLDM6×His Y140H + VCZ (PDB ID: 7RY8); (**c**) ScLDM6×His I371T + VCZ (PDB ID: 7RY9); (**d**) ScLDM6×His Y140H Y471T + VCZ (PDB ID: 7RYB); and (**e**) ScLDM6×His I371T + ITC (PDB ID: 7RYA). For protein: O atoms in red, N atoms in blue, S atoms in mustard. For VCZ and ITC: carbon in purple, nitrogen in blue, and fluorine or chlorine in white. The surface net reflects the color of adjacent protein residues. SEC, substrate entry channel. AO, accessory opening.

**Figure 8:**
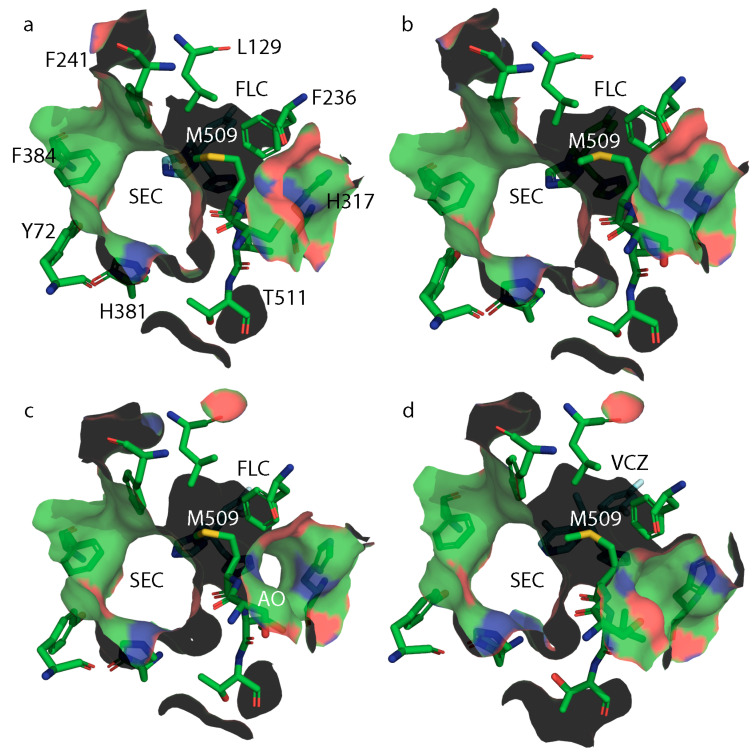
Surface structures at the neck of the substrate entry channel in ScLDM6×His and its mutants in complex with short-tailed azoles. (**a**) ScLDM6×His (WT) and with FLC (PDB ID: 4WMZ); (**b**) ScLDM6×His Y140H with FLC (PDB ID: 4ZE3); (**c**) ScLDM6×His Y140F with FLC (PDB ID: 4ZDZ); and (**d**) ScLDM6×His Y140F with VCZ (PDB: 4ZE0). For protein: carbon in green, oxygen in red, nitrogen in blue, sulfur in mustard. For FLC and VCZ: carbon in purple. The surface reflects the color of adjacent protein atoms. SEC, substrate entrance channel. AO, accessory opening.

Analysis of the surface structure of wild-type ScLDM6×His in complex with VCZ (Figure 7a) indicates a constriction of the neck of SEC in the presence of this short-tailed ligand, due to the different orientation of M509, plus the appearance of an accessory opening (AO) beside the proton channel. This opening is not found in structures of ScLDM6×His (WT) in complex with FLC (Figure 8a, PDB ID: 4WMZ), VT-1161 (PDB ID: 5UL0), ITC (PDB ID: 5EQB), and PCZ (PDB ID: 6E8Q), or ScLDM6×His Y140F in complex with VCZ (Figure 8d, PDB ID: 4ZE0), or ScLDM6×His I471T in complex with ITC (Figure 7e, PDB ID: 7RYA). Furthermore, for ScLDM6×His I471T in complex with VCZ (Figure 7b, PDB ID: 7RY9), the neck of the SEC is completely closed, and the AO is also present. This implies that the distant I471T mutation could modify the accessibility of some ligands to the LDM active site. In contrast, both ScLDM6×His Y140H and ScLDM6×His Y140H I471T in complex with VCZ have no AO, but have an open SEC of a similar cross-sectional area at its narrowest point, i.e., the opening bounded by the M509 methyl and H381 imidazole nitrogen, and the F241 benzene ring and T511 sidechain methyl is ~48 Å^2^ for ScLDM6×His Y140H in complex with VCZ, and ~49 Å^2^ for ScLDM6×His Y140H I471T in complex with VCZ. ScLDM6×His in complex with ITC shows that the long tail of ITC occupies the SEC, with M509 adopting a conformation closely matching that found in ScLDM6×His Y140F and ScLDM6×His Y140F I471T complexed with VCZ, but not ScLDM6×His or ScLDM6×His I471T complexed with VCZ. These results suggest a critical role of the water-mediated hydrogen bond network between the VCZ 5-fluoropyrimidine ring and the main chain carbonyl of M509 in defining access to the active site. The absence of the SEC neck constriction in the structures shown in Figure 8 is consistent with this interpretation, i.e., ScLDM6×His, (PDB ID: 4WMZ), ScLDM6×His Y140H (PDB ID: 4ZE3), and ScLDM6×His Y140F (PDB ID: 4ZDZ) in complex with FLC, as well as ScLDM6×His Y140F in complex with VCZ (PDB ID: 4ZE0). None of these structures enable the formation of a water-mediated hydrogen bond network between a FLC or VCZ nitrogen and the main chain carbonyl of M509 that would alter the conformation of both the main chain and sidechain of this amino acid. In contrast, maintenance of water-mediated hydrogen bonding with the S382 main chain carbonyl appears to constrain the conformation of the neighboring H381 residue that significantly contributes to the structure of the neck of the SEC. The appearance of the AO seems more complicated and requires either small (Figure 8c, ScLDM6×His Y140F in complex with FLC) or larger (Figure 7a,b, ScLDM6×His or ScLDM6×His I471T in complex with VCZ) changes in conformation of M509, plus a modified conformation of the H317 sidechain in helix I involved in the proton channel.

## 4. Discussion

We have engineered the model yeast *S. cerevisiae* to overexpress the ScLDM Y140H and I471T mutations, either alone or in combination, as surrogates to provide insight into the target-mediated susceptibility of the azole resistant Darlington strain of *C. albicans*. This approach has demonstrated that the ScLDM I471T mutation (equivalent to I471T in CaLDM) confers on the Y140H mutation (equivalent to Y132H in CaLDM) a synergistic reduction in the susceptibility of host *S. cerevisiae* cells to the short-tailed azoles FLC and VCZ, but not to the medium-tailed azole VT-1161, or the long-tailed azoles ITC and PCZ. Our results imply that the strong resistance to FLC and VCZ and the weaker resistance to VT-1161 in the *C. albicans* Darlington strain are dominated by the overexpression of the CaLDM Y132H I471T. The effect of a Cdr1-specific inhibitor on the modest resistance to the long-tailed azoles ITC and PCZ shows that the expression of the drug efflux pump in the control sensitive SC5314 strain and its overexpression in the Darlington strain make significant additional contributions to the resistance. This finding is corroborated by Albertson et al. [36] who showed that the Darlington strain produced significantly more *ERG11* (then referred to as *ERG16*) and *CDR1* mRNA than azole-susceptible control strains, whereas *MDR1* (also called *BEN^r^*) mRNA was expressed at levels comparable to azole-susceptible strains. The mild resistance of Darlington cells to the polyene AMB observed here has been reported previously, and appears consistent with a significantly increased production of ergosterol [51], due to the *ERG11* overexpression detected in this strain [36].

In CaLDM of the Darlington strain and the surrogate recombinant ScLDM6×His, the I471T mutation is adjacent to the heme cofactor in proximity of helices C and I, and below the FSL located on the proximal side of the heme. Several mutations in the FSL are known to confer azole resistance, but are too far from the active site pocket to have a direct impact on azole binding, which occurs on the distal side of the heme adjacent to helix I [2,21,45]. The FSL is part of the proposed site of interaction with the cognate NADPH-cytochrome P450 reductase that shuttles electrons into the active site of LDM [63]. An increase in catalytic efficiency caused by resistance-conferring mutations that occur in this region has been suggested to result from enhanced interaction with the cognate NADPH-cytochrome P450 reductase [51]. Detergent solubilized CaLDM containing the I471T single-mutation is reported to have a 2.4-fold higher affinity for the endogenous substrate lanosterol than the wild-type protein, and a 2-fold higher demethylase reaction velocity in the presence of FLC [40]. If the I471T mutation affects drug binding due to competition with substrate, Type I binding experiments that assess the extent and affinity of lanosterol binding can be expected to be informative. If the I471T mutation contributes to azole resistance via an increase in catalytic efficiency, a biochemical assay that measures the impact of lanosterol on LDM activity and antifungal susceptibility using the substrate BOMCC [64] and crude membranes co-expressing cognate NADPH-cytochrome P450 reductase with ScErg11-6×His I471T or CaLDM I471T may provide experimental conditions more representative of the in vivo setting. Measurement of the modulation of enzyme catalysis by test azoles may detect differences in drug affinity that are not seen with the Type I or Type II binding studies, and provide more meaningful information about how the mutation influences enzyme-drug interactions. Correlation of these results with the expression data and whole cell drug susceptibility assays will help identify structure-activity relationships for the test azoles. 

Cells containing the Y140H mutation in ScLDM6×His show a significant but modest (2-fold) resistance to VT-1161, but the I471T mutation does not amplify the resistance synergistically, unlike for FLC and VCZ (Table 1). The tetrazole VT-1161 contains a slightly longer tail than FLC and VCZ, but a shorter tail than ITC and PCZ (Figure 1). The long-tailed azoles ITC and PCZ do not have a tertiary alcohol within the linker region and, as such, their binding was unaffected by the Y140H mutation, and did not confer resistance to azole treatment (Figure 1 and Table 1). The Y140H I471T double mutation in ScLDM6×His also failed to confer significant resistance on *S. cerevisiae* cells to ITC and PCZ, or to enhance the resistance to VT-1161. This suggests that the tail substituents not only block movement of M509, but also provide sufficient interaction with the walls of the SEC to overcome the combined effect of the Y140H and I471T mutations in the active site, as well as the slightly weaker interaction of the tetrazole with the heme. However, mutations that occur near the mouth of the SEC, such as G54S/W/E in *A. fumigatus* CYP51A (equivalent to *S. cerevisiae* G73S/W/E), have been shown to confer resistance to the long-tailed azoles [65,66]. This is likely due to such mutations preventing access of ITC to the active site or its stable binding. 

The ScLDM6×His Y140H I471T mutant provides a surrogate X-ray crystal structure for the clinically relevant Darlington LDM mutant of *C. albicans*, and for the LDM Y131F I475T mutant from the phytopathogen *P. pachyrhizi*. Comparison of the X-ray crystal structures of wild-type, Y140H, and/or I471T mutant ScLDM6×His in complex with VCZ or ITC showed that the tilt of the heme is not changed by the mutations, despite a loss of the polar interaction between Y140 and the heme ring C propionate, a water-mediated interaction between the VCZ hydroxyl group and the heme ring D propionate, and a hydrogen bond between Y126 and the heme ring D propionate. Due to the movement of VCZ towards helix I and the mutated histidine, the Y140H mutation ablates a key water-mediated hydrogen bond network involving the VCZ 5-fluoropyrimidine, the main chain amide of S382, and the main chain carbonyls of M509. While we were unable to differentiate the ScLDM6×His wild-type and I471T structures from the mutation at I471, we detected spectral changes consistent with modification of the electronic environment of the heme. Unfortunately, the structures obtained do not elucidate how the Y140H and I471T mutations interact to confer enhanced resistance to short-tailed azoles, i.e., the double mutation maintains enzyme function in the presence of the short-tailed triazole inhibitors FLC and VCZ. This effect is overcome by long-tailed triazole inhibitors ITC and PCZ, and substantially by the medium-tailed azole inhibitor VT-1161, none of which form a water-mediated hydrogen bond network with the main chain carbonyl of M509 and the main chain nitrogen of S382, which has the near neighbors H381 and F384, plus F241, that are important for defining the dimensions of the neck of the SEC. 

Hargrove et al. recently demonstrated that the efficient binding to human CYP51 of the substrate lanosterol can be achieved by using site-directed mutagenesis to block the proton transfer into the active site required for catalysis [47]. Upon binding of lanosterol in the active site, the helix C residue structural equivalent to K151 in ScLDM loses its ionic interaction with the heme ring C propionate, and presents its sidechain amino group above the enzyme surface. This is expected to enable the efficient binding of the cognate NADPH-cytochrome P450 reductase. The ScLDM I471T mutation is therefore predicted to reduce hydrophobic interactions within the environment involving sidechain methyl groups of V154, L158, and the β and the ε methylene carbons of K151. The resultant increase in conformational flexibility in helix C is expected to enable more efficient cognate reductase binding, while altered F-G helix conformation (F236-F241) should enhance lanosterol entry into the active site. This should reduce opportunities for the competitive binding of the short-tailed azoles FLC and VCZ, potentiating the impact of the Y140F/H mutations that open the neck of the SEC at mouth of the active site. This agrees with results obtained with CaLDM6×His, in which co-expression in *S. cerevisiae*, with its cognate reductase, conferred increased resistance to FLC and VCZ, but not to VT-1161, ITC, or PCZ [51]. Collectively, our results suggest that the incorporation of a suitable medium-length tail in the design of next-generation azoles can exploit interactions with the walls of the SEC in human and plant fungal pathogen SDMs, to counter mutations in the active site that increase competition with the enzyme substrates lanosterol or eburicol.

## Figures and Tables

**Table 1:** Susceptibilities to azoles of test and control yeast strains.

Strain	MIC_80_						
	FLC (µM)	VCZ (nM)	VT-1161 (nM)	ITC (nM)	PCZ (nM)	MCF (nM)	AMB (μM)
**AD2Δ**	1.33 (0.06)	37.50 (0.50)	28.75 (3.92)	73.50 (0.65)	160.40 (21.40)	173.80 (11.07)	2.18 (0.27)
**AD3Δ**	3.64 (0.37)	93.33 (5.83)	75.40 (2.91)	143.40 (0.46)	423.30 (29.91)	145.80 (17.05)	2.34 (0.28)
**Y140H**	7.40 (0.48)	163.70 (5.24)	115.60 (10.59)	112.10 (3.03)	330.00 (15.10)	166.00 (12.80)	2.19 (0.28)
**I471T**	1.88 (0.60)	74.00 (0.58)	34.63 (0.55)	102.10 (4.87)	269.50 (22.86)	164.40 (13.03)	2.26 (0.34)
**Y140H + I471T**	11.50 (0.70)	249.60 (3.25)	67.60 (1.63)	107.90 (1.07)	264.80 (23.13)	162.40 (13.03)	2.14 (0.25)

Data is expressed as the mean concentration required to inhibit 80% of yeast growth, compared with a non-drug control (MIC_80_, *n* = 3). Values in parenthesis indicate ± standard error of the mean.

**Table 2:** Susceptibilities to azoles of test and control yeast strains taking into account enzyme expression.

MIC_80_/Relative Amount of ScLDM6×His					
Strain (Relative Expression)	FLC (µM)	VCZ (nM)	VT-1161 (nM)	ITC (nM)	PCZ (nM)
**AD3Δ (1)**	3.64	93.3	75.4	143	423
**Y140H (0.74)**	10 (2.8×)	221 (2.4×)	157 (2.1×)	151 (1.1×)	445 (1.1×)
**I471T (0.51)**	3.69 (1.0×)	145 (1.6×)	67.9 (0.90×)	200 (1.4×)	527 (1.3×)
**Y140H+I471T (0.41)**	28.0 (7.7×)	609 (6.5×)	165 (2.2×)	263 (1.8×)	646 (1.5×)

Values from Table 1 are normalized relative to the amount of ScLDM6×His in crude membrane preparations from each strain (parenthesis in bold), detected using an anti-His tag antibody relative to strain AD3Δ (Figure 2b). The values in parenthesis indicate the fold-resistance of each recombinant strain based on the relative amount of recombinant enzyme in crude membrane preparations compared with strain AD3Δ.

## Data Availability

Refined X-ray crystal structures with accession codes 7RY8, 7RY9, 7RYA, and 7RYB have been lodged in the Protein Data Bank.

## Supplementary Materials

The following are available online at https://www.mdpi.com/article/10.3390/jof7110897/s1, Figure S1: Mass spectrometry analysis of proteolytic fingerprints of ScLDM6×His, Figure S2: Difference spectra showing type II binding of VCZ and ITC to wild-type (AD3Δ) ScErg11p6×His and ScErg11p6×His I471T mutant, Figure S3: OMIT maps for the binding VCZ to (a) ScLDM6×His I471T and (b) ScLDM6×His Y140H + I471T, Figure S4: Binding of ITC to ScLDM6×His I471T, Table S1: Yeast strains used in this study, Table S2: Oligonucleotide primers used in the study, Table S3: Data collection and refinement statistics for ScLDM6×His Y140H in complex with VCZ, ScLDM6×His I471T in complex with ITC and VCZ, and ScLDM6×His Y140H + I471T in complex with VCZ. Table S4: Azole binding to affinity purified wild-type (AD3Δ) ScLDM6×His and ScLDM6×His I471T mutant.
