# Supplementary material for: Structural Insights into the Azole Resistance of the Candida albicans Darlington Strain Using Saccharomyces cerevisiae Lanosterol 14α-Demethylase as a Surrogate
Source: J Fungi (Basel). 2021 Oct 24;7(11):897. doi: 10.3390/jof7110897 (PMC8621857; doi:10.3390/jof7110897)
Supplement: Supplementary file 1 [file jof-07-00897-s001.zip › jof-1400024-supplementary.pdf]

Supplementary materials: Graham et al.

Table S1. Yeast strains used in this study.

Strain	Genotype	Reference
**ADΔ**	MATα PDR1-3 Δyor1::hisG Δsnq2::hisG Δpdr3::hisG Δpdr10::hisG Δpdr11::hisG Δycf1::hisG Δpdr5::hisG Δpdr15::hisG Δura3 ΔhisAD124567 Δpdr5::hisG Δpdr15::hisG, Δura3::dpl200	Lamping *et al.* (2007) [1]
**AD2Δ**	ADΔ, Δhis1::dpl200	Sagatova *et al.* (2015) [2]
**AD3Δ ScErg11**	AD2Δ, Δpdr5::ScERG11-URA3, Δerg11::HIS1	Sagatova *et al.* (2015) [2]
**AD2Δ ScErg11 Y140H**	AD2Δ, Δpdr5::ScERG11_Y140H-URA3	This study
**AD3Δ ScErg11 Y140H**	AD2Δ Δpdr5::ScERG11_Y140H, Δerg11::HIS1	This study
**AD2Δ ScErg11 I471T**	AD2Δ, Δpdr5::ScERG11_I471T-URA3	This study
**AD3Δ ScErg11 I471T**	AD2Δ Δpdr5::ScERG11_I471T, Δerg11::HIS1	This study
**AD2Δ ScErg11 Y140H I471T**	AD2Δ, Δpdr5::ScERG11_Y140H+I471T-URA3	This study
**AD3Δ ScErg11 Y140H I471T**	AD2Δ Δpdr5::ScERG11_Y140H+I471T, Δerg11::HIS1	This study

Table S2. Oligonucleotide primers used in the study

Creation of LDM6×His I471T

Forward primer ScErg11p_I471T_f
GGTGGTGGTAGACACAGATGT**ACC**GGTGAACACTTTGCTTAC

Reverse primer ScErg11p_I471T_r
GTAAGCAAAGTGTTCACC**GGT**ACATCTGTGTCTACCACCACC

Creation of LDM6×His Y140H

Forward primer ScErg11p_Y140H_f

AAGGTGTTATT**CAT**GATTGTCCAAATTC

Reverse primer ScErg11p_Y140H_r

TTGGACAATC**ATG**AATAACACCTTTACC

Table S3. Data collection and refinement statistics for ScLDM6×His Y140H in complex with VCZ, ScLDM6×His I471T in complex with ITC and VCZ, and ScLDM6×His Y140H + I471T in complex with VCZ

Protein	Y140H VCZ	I471T VCZ	I471T ITC	Y140H+I471T VCZ
PDB ID	7RY8	7RY9	7RYA	7RYB
Space group	$P\ 1\ 2_1\ 1$	$P\ 1\ 2_1\ 1$	$P\ 1\ 2_1\ 1$	$P\ 1\ 2_1\ 1$
Resolution range (Å)	39.99 – 1.98 (2.03 – 1.98)	33.15 – 2.4 (2.49 – 2.40)	33.42 – 2.1 (2.18 – 2.10)	33.75 - 2.9 (3.00 – 2.90)
Unit cell axes (Å)	a = 77.79, b = 66.86, c = 80.88	a = 76.79, b = 66.31, c = 80.73	a = 77.53, b = 66.70, c = 80.58	a = 79.18, b = 67.30, c = 81.33
Total reflections	293230	62937	90476	36666
Unique reflections	57296	31547	45698	18602
Completeness (%)	99.8 (99.90)	99.64 (99.90)	95.73 (85.33)	98.21
<I/σ(I)>	7.2 (0.98)	13.19 (2.01)	13.86 (2.39)	9.00 (1.43)
Wilson B-factor	43.10	38.91	27.94	54.02
R_{merge}	0.147 (3.302)	0.044 (0.380)	0.037 (0.285)	0.067 (0.566)
CC(1/2)	0.996 (0.317)	0.998 (0.719)	0.998 (0.836)	0.992 (0.753)
Refinement				
R_{work}	0.209 (0.362)	0.199 (0.282)	0.197 (0.259)	0.201 (0.294)
R_{free}	0.234 (0.357)	0.237 (0.307)	0.235 (0.308)	0.252 (0.353)
Number of reflections	57118 (5548)	31538 (3119)	45680 (4038)	18592 (1830)
Number of atoms in model				
Protein	4478	4262	4280	4228
Ligand	96	81	92	68
Water molecules	123	67	196	1
Deviation from ideal bond lengths (Å)	0.007	0.008	0.008	0.009
Deviation from ideal bond angles (°)	0.84	0.93	0.90	1.02
Ramachandran analysis (%)				
Preferred	97.14	95.62	97.15	92.90
Allowed	2.67	4.00	2.66	6.72
Residues in disallowed regions	0.19	0.38	0.19	0.38

Statistics for the highest resolution shell shown in parentheses. One crystal was used per structure. R_{free} was calculated using a test set composed of 5% of data.

This research was undertaken on the MX1 beamline at a wavelength of 0.954 Å at the Australian Synchrotron, part of ANSTO [3].

Confirmation of LDM mutations by mass spectrometry of tryptic or chymotryptic fingerprints

Mass spectrometry (MS-MS) analysis of tryptic or chymotryptic fragments of the 62 kDa protein band gave protein sequence coverage of at least 54% of the ScLDM6×His primary sequence and detected the peptides showing the expected Y140H and I471T mutations (**Figure S1**).

A.

```
11   MSATKSIVGE  ALEYVNIGLS  HFLALPLAQR  ISLIIIIPFI
441  YNIVWQLLYS  LRKDRPPLVF  YWIPWVGSAV  VYGMKPYEFF
 81  EECQKKYGDI  FSFVLLGRVM  TVYLGPKGHE  FVFNAKLADV
121  SAEAAYAHLT  TPVFGKGVIH  DCPNSRLMEQ  KKFVKGALTK
161  EAFKSYVPLI  AEEVYKYFRD  SKNFRLNERT  TGTIDVMVTQ
201  PEMTIFTASR  SLLGKEMRAK  LDTDFAYLYS  DLDKGFTPIN
241  FVFPNLPLEH  YRKRDHAQKA  ISGTYMSLIK  ERRKNNDIQD
281  RDLIDSLMKN  STYKDGVKMT  DQEIANLLIG  VLMGGQHTSA
321  ATSAWILLHL  AERPDVQQEL  YEEQMRVLDG  GKKELTYDLL
361  QEMPLLNQTI  KETLRMHHPL  HSLFRKVMKD  MHVPNTSYVI
401  PAGYHVLVSP  GYTHLRDEYF  PNAHQFNIHR  WNNDSASSYS
441  VGEEVDYGFG  AISKGVSSPY  LPFGGGRHRC  IGEHFAYCQL
481  GVLMSIFIRT  LKWHYPEGKT  VPPPDFTSMV  TLPTGPAKII
521  WEKRNPEQKI  GGRHHHHHH
```

B.

```
1    MSATKSIVGE  ALEYVNIGLS  HFLALPLAQR  ISLIIIIPFI
41   YNIVWQLLYS  LRKDRPPLVF  YWIPWVGSAV  VYGMKPYEFF
81   EECQKKYGDI  FSFVLLGRVM  TVYLGPKGHE  FVFNAKLADV
121  SAEAAYAHLT  TPVFGKGVIY  DCPNSRLMEQ  KKFVKGALTK
161  EAFKSYVPLI  AEEVYKYFRD  SKNFRLNERT  TGTIDVMVTQ
201  PEMTIFTASR  SLLGKEMRAK  LDTDFAYLYS  DLDKGFTPIN
241  FVFPNLPLEH  YRKRDHAQKA  ISGTYMSLIK  ERRKNNDIQD
281  RDLIDSLMKN  STYKDGVKMT  DQEIANLLIG  VLMGGQHTSA
321  ATSAWILLHL  AERPDVQQEL  YEEQMRVLDG  GKKELTYDLL
361  QEMPLLNQTI  KETLRMHHPL  HSLFRKVMKD  MHVPNTSYVI
401  PAGYHVLVSP  GYTHLRDEYF  PNAHQFNIHR  WNNDSASSYS
441  VGEEVDYGFG  AISKGVSSPY  LPFGGGRHRC  TGEHFAYCQL
481  GVLMSIFIRT  LKWHYPEGKT  VPPPDFTSMV  TLPTGPAKII
521  WEKRNPEQKI  GGRHHHHHH
```

C.

```
1    MSATKSIVGE  ALEYVNIGLS  HFLALPLAQR  ISLIIIIPFI
41   YNIVWQLLYS  LRKDRPPLVF  YWIPWVGSAV  VYGMKPYEFF
81   EECQKKYGDI  FSFVLLGRVM  TVYLGPKGHE  FVFNAKLADV
121  SAEAAYAHLT  TPVFGKGVIH  DCPNSRLMEQ  KKFVKGALTK
161  EAFKSYVPLI  AEEVYKYFRD  SKNFRLNERT  TGTIDVMVTQ
201  PEMTIFTASR  SLLGKEMRAK  LDTDFAYLYS  DLDKGFTPIN
241  FVFPNLPLEH  YRKRDHAQKA  ISGTYMSLIK  ERRKNNDIQD
281  RDLIDSLMKN  STYKDGVKMT  DQEIANLLIG  VLMGGQHTSA
321  ATSAWILLHL  AERPDVQQEL  YEEQMRVLDG  GKKELTYDLL
361  QEMPLLNQTI  KETLRMHHPL  HSLFRKVMKD  MHVPNTSYVI
401  PAGYHVLVSP  GYTHLRDEYF  PNAHQFNIHR  WNNDSASSYS
441  VGEEVDYGFG  AISKGVSSPY  LPFGGGRHRC  TGEHFAYCQL
481  GVLMSIFIRT  LKWHYPEGKT  VPPPDFTSMV  TLPTGPAKII
521  WEKRNPEQKI  GGRHHHHHH
```

Figure S1: Mass spectrometry analysis of proteolytic fingerprints of ScLDM6×His. Tryptic (A and C) and chymotryptic (B) digests of the 62-kDa protein band excised from the SDS-PAGE gel profiles of crude membrane samples were analysed using Orbitrap-based mass spectrometry at the University of Otago, Department of Biochemistry Centre for Protein Research. Protein bands were digested using the DigestPro MSi (Intavis AG, Cologne, Germany) robotic workstation according to an established protocol [4]. The Mascot search engine was used to search the data obtained from the SWISS-PROT amino acid sequence database. The amino acid sequences of protein fragments from the ScLDM6×His mutants identified in the proteolytic digests are highlighted in bold. **(A)** The ScLDM6×His Y140H sample showed 54% coverage of the primary sequence. The Y140H mutation is highlighted in green. **(B)** The ScLDM6×His I471T sample showed 94.8% coverage of the primary sequence. The I471T mutation is highlighted in blue. **(C)** The ScLDM6×His Y140H + I471T sample showed 98.9% coverage. The Y140H and the I471T mutation are highlighted in green and blue, respectively.

Assessment of in vitro binding affinity of test azoles to Ni-NTA affinity purified ScLDM6×His

The affinity of the test azoles for Ni-NTA affinity purified ScLDM6×His was measured using an *in vitro* azole-binding assay (**Table S2**). We previously reported there were no significant differences between the K_d values (0.5 μM range) for wild-type and Y140F/H mutants of ScLDM6×His binding to FLC, VCZ, ITC, and PCZ [5]. Because of lower stability of the detergent solubilised ScLDM6×His Y140H I471T, *in vitro* drug binding assays using the short-tailed azole VCZ and the long-tailed azole ITC were conducted for control wild-type ScLDM6×His (AD3Δ) and ScLDM6×His I471T preparations only (**Figure 4, figure S2 and Table S2**). Both ITC and VCZ showed tight type II binding to wild-type and I471T mutant ScLDM6×His, with $[\text{Azole}]_{0.5}$ values of ~0.75 μM in the presence of 2 μM functional enzyme and K_d values in the 0.5 μM range. Other studies failed to find a correlation between whole cell drug susceptibility assays and azole affinity for purified LDM, including CaLDM containing the Y132H and I471T mutations [5, 6]. Sagatova *et al.* (2016) hypothesised that this may be due to the formation of near stoichiometric LDM-azole complexes that limit accuracy in the calculation of azole affinities [5]. It has also been shown that detergent solubilisation of *A. fumigatus* alters the ligand binding properties of the enzyme [7]. This is unsurprising as the X-ray crystal structure of full-length ScLDM6×His revealed rigid anchorage in the lipid bilayer of the mouth of the substrate entry channel that enables substrates and azole drugs to reach the

buried active site (PDB ID: 4LXJ) [8, 9]. Detergent solubilisation of ScLDM6×His could perturb this entry route to the active site and may explain the apparent lack of correlation between the results of whole cell drug susceptibility assays and *in vitro* azole binding assays. While the modest Hill numbers (1.55 – 1.85) obtained from the binding curves (**Figure 4**) might indicate cooperative binding between ScLDM6×His monomers, Sagatova *et al*. (2016) hypothesised that such values are more likely to represent multiple interactions between the azole and the internal active site of ScLDM6×His [5]

.

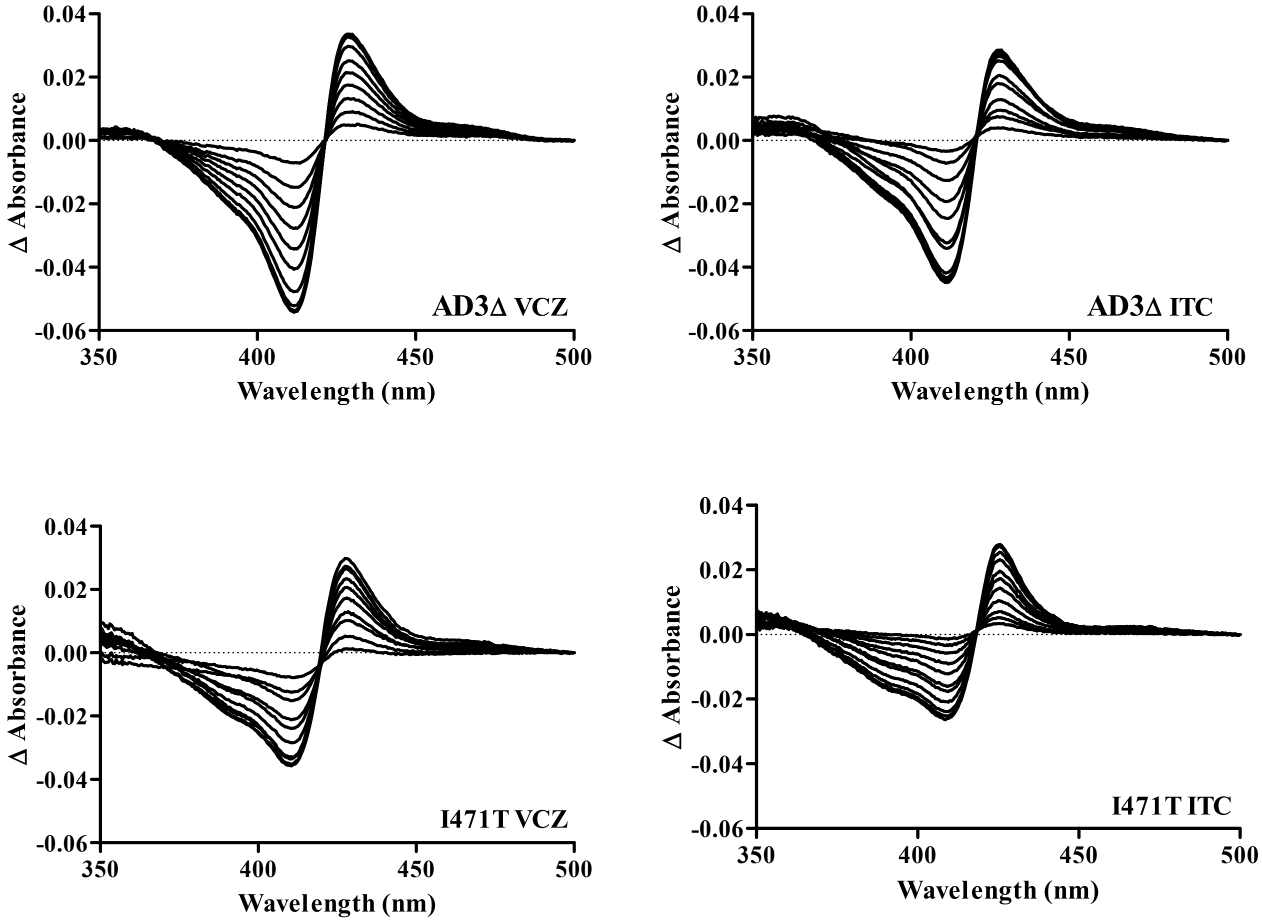

Figure S2. Difference spectra showing type II binding of VCZ and ITC to wild-type (AD3Δ) ScErg11p6×His and ScErg11p6×His I471T mutant. Difference spectra were obtained by titrating a 0.5 mL sample of 2 μM functional ScErg11p6×His as described in Materials and Methods.

Table S4: Azole binding to affinity purified wild-type (AD3Δ), ScLDM6×His and ScLDM6×His I471T mutant.

ScLDM6×His	Azole	ΔA_{max}	$[Azole]_{0.5}$	K_d (μM)	Hill number
AD3Δ	VCZ	0.10 (0.004)	0.78 (0.06)	0.62 (0.11)	1.85 (0.23)
	ITC	0.08 (0.006)	0.60 (0.07)	0.43 (0.12)	1.61 (0.22)
I471T	VCZ	0.07 (0.003)	0.75 (0.05)	0.58 (0.10)	1.88 (0.23)
	ITC	0.06 (0.001)	0.79 (0.10)	0.70 (0.16)	1.55 (0.19)

Values in brackets represent ± standard errors of the mean.

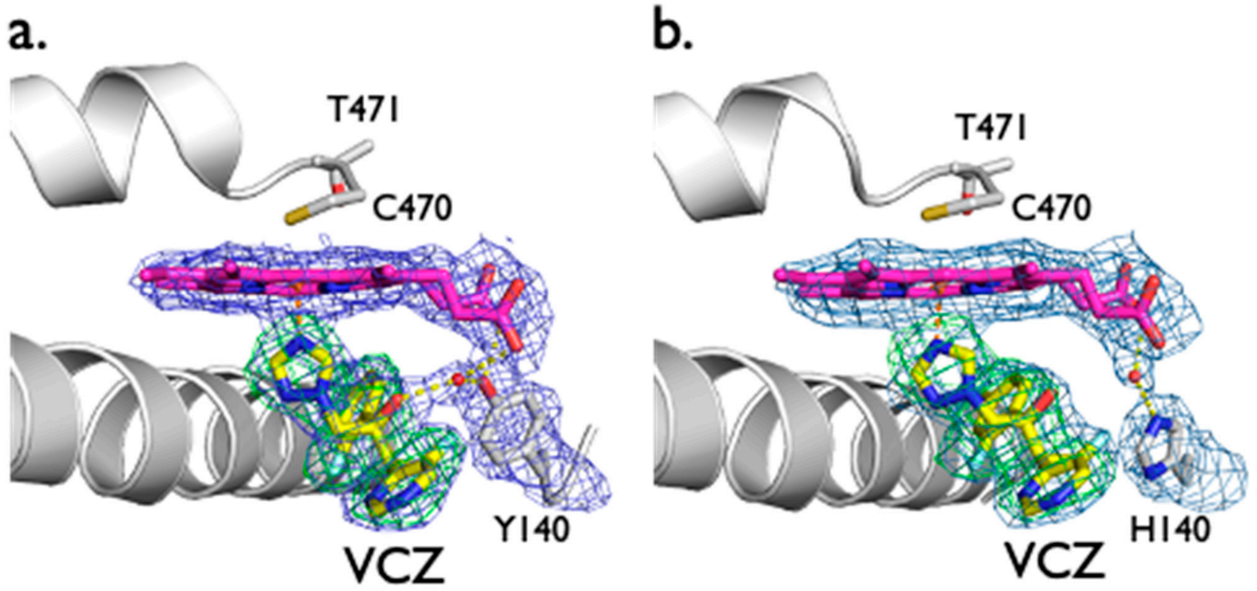

Figure S3. OMIT maps for the binding VCZ to (a) ScLDM6×His I471T and (b) ScLDM6×His Y140H + I471T. VCZ is shown as sticks, C atoms yellow, N atoms blue, O atoms red, F atoms pale blue. The heme cofactor is shown with C atoms in magenta. Electron density is shown for VCZ, heme, water molecules, and the site of the native Y140 residue and H140 mutation. The $2F_o - F_c$ electron density map (blue) is contoured at 1σ and the $Fo - Fc$ map (green) is contoured at 3σ. OMIT maps were generated by Phenix and visualised in PyMOL. The $Fo - Fc$ map was calculated using F_{calc} from the refinement process with no ligand in the active site. The $2F_o - F_c$ map was calculated following final refinement after modelling VCZ in the active site. Water molecules are shown as red spheres. Hydrogen bonds are shown as dashed yellow lines.

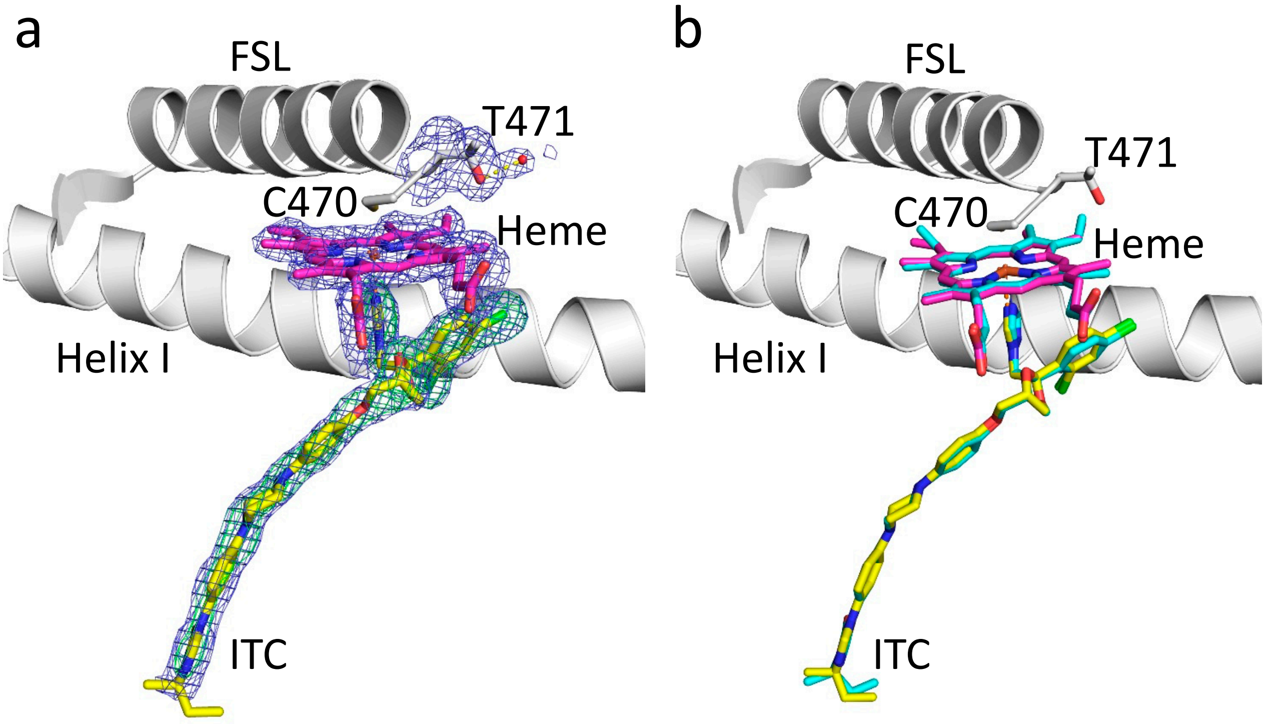

Figure S4. Binding of ITC to ScLDM6×His I471T. (**a**) OMIT map is shown for ITC, the heme, and T471 with associated water molecule. ITC is shown as sticks, C atoms in yellow, N atoms in blue, O atoms in red, Cl atoms in green. The heme is shown with C atoms in magenta. The $2F_o - F_c$ electron density map (blue) is contoured at 1σ and the $Fo - Fc$ map (green) is contoured at 3σ. OMIT maps were generated as in **Figure S3**. Water molecules are shown as red spheres. Hydrogen bonds are shown as dashed yellow lines. (**b**) Structural alignment of ScLDM6×His I471T in complex with ITC (ITC with C atoms in yellow, haem with C atoms in magenta) and wild-type ScLDM6×His in complex with ITC (ITC and haem in cyan) (PDB ID: 5EQB). The position of ITC and the heme is unchanged between the ScLDM6×His wild-type and I471T mutant structures.

References:

1. Lamping, E.; Monk, B. C.; Niimi, K.; Holmes, A. R.; Tsao, S.; Tanabe, K.; Niimi, M.; Uehara, Y.; Cannon, R. D., Characterization of three classes of membrane proteins involved in fungal azole resistance by functional hyperexpression in *Saccharomyces cerevisiae*. *Eukaryot Cell* **2007,** *6* (7), 1150-65.
2. Sagatova, A. A.; Keniya, M. V.; Wilson, R. K.; Monk, B. C.; Tyndall, J. D., Structural Insights into Binding of the Antifungal Drug Fluconazole to *Saccharomyces cerevisiae* Lanosterol 14alpha-Demethylase. *Antimicrob Agents Chemother* **2015,** *59* (8), 4982-9.
3. McPhillips, T. M.; McPhillips, S. E.; Chiu, H. J.; Cohen, A. E.; Deacon, A. M.; Ellis, P. J.; Garman, E.; Gonzalez, A.; Sauter, N. K.; Phizackerley, R. P.; Soltis, S. M.; Kuhn, P., Blu-Ice and the Distributed Control System: software for data acquisition and instrument control at macromolecular crystallography beamlines. *J Synchrotron Radiat* **2002,** *9* (Pt 6), 401-6.
4. Shevchenko, A.; Jensen, O. N.; Podtelejnikov, A. V.; Sagliocco, F.; Wilm, M.; Vorm, O.; Mortensen, P.; Shevchenko, A.; Boucherie, H.; Mann, M., Linking genome and proteome by mass spectrometry: large-scale identification of yeast proteins from two dimensional gels. *Proc Natl Acad Sci U S A* **1996,** *93* (25), 14440-5.
5. Sagatova, A. A.; Keniya, M. V.; Wilson, R. K.; Sabherwal, M.; Tyndall, J. D.; Monk, B. C., Triazole resistance mediated by mutations of a conserved active site tyrosine in fungal lanosterol 14alpha-demethylase. *Sci Rep* **2016,** *6*, 26213 doi: 10.1038/srep26213.
6. Park, H. G.; Lee, I. S.; Chun , Y. J.; Yun, H. C.; Johnston, J. B.; Montellano, P. R.; Kim, D., Heterologous expression and characterization of the sterol 14alpha-demethylase CYP51F1 from *Candida albicans*. *Arch Biochem Biophys* **2011,** *509* (1), 9-15.
7. Warrilow, A. G.; Parker, J. E.; Price, C. L.; Nes, W. D.; Kelly, S. L.; Kelly, D. E., In Vitro Biochemical Study of CYP51-Mediated Azole Resistance in *Aspergillus fumigatus*. *Antimicrob Agents Chemother* **2015,** *59* (12), 7771-8.
8. Monk, B. C.; Tomasiak, T. M.; Keniya, M. V.; Huschmann, F. U.; Tyndall, J. D.; O'Connell, J. D., 3rd; Cannon, R. D.; McDonald, J. G.; Rodriguez, A.; Finer-Moore, J. S.; Stroud, R. M., Architecture of a single membrane spanning cytochrome P450 suggests constraints that orient the catalytic domain relative to a bilayer. *Proc Natl Acad Sci U S A* **2014,** *111* (10), 3865-70.
9. Pochapsky, T. C., Examining how enzymes self-organize in a membrane. *PNAS* **2014,** *111* (1091-6490 (Electronic)), 3659–3660.
